# Fibroblasts generate topographical cues that steer cancer cell migration

**DOI:** 10.1126/sciadv.ade2120

**Published:** 2023-08-16

**Authors:** Francesco Baschieri, Abigail Illand, Jorge Barbazan, Olivier Zajac, Clémence Henon, Damarys Loew, Florent Dingli, Danijela Matic Vignjevic, Sandrine Lévêque-Fort, Guillaume Montagnac

**Affiliations:** ^1^ Inserm U1279, Gustave Roussy Institute, Université Paris-Saclay, Villejuif, France.; ^2^ Université Paris Saclay, CNRS, Institut des sciences moléculaires d’Orsay, UMR8214, Orsay, France.; ^3^ Translational Medical Oncology Group (ONCOMET), Health Research Institute of Santiago de Compostela (IDIS), Santiago de Compostela, Spain.; ^4^ Institut Curie, UMR144, PSL Research University, Centre Universitaire, Paris, France.; ^5^ Inserm U981, Gustave Roussy Institute, Université Paris-Saclay, Villejuif, France.; ^6^ Institut Curie, PSL Research University, Centre de Recherche, Laboratoire de Spectrométrie de Masse Protéomique, Paris, France.

## Abstract

Fibroblasts play a fundamental role in tumor development. Among other functions, they regulate cancer cells’ migration through rearranging the extracellular matrix, secreting soluble factors, and establishing direct physical contacts with cancer cells. Here, we report that migrating fibroblasts deposit on the substrate a network of tubular structures that serves as a guidance cue for cancer cell migration. Such membranous tubular network, hereafter called tracks, is stably anchored to the substrate in a β5-integrin–dependent manner. We found that cancer cells specifically adhere to tracks by using clathrin-coated structures that pinch and engulf tracks. Tracks thus represent a spatial memory of fibroblast migration paths that is read and erased by cancer cells directionally migrating along them. We propose that fibroblast tracks represent a topography-based intercellular communication system capable of steering cancer cell migration.

## Teaser

The migration path of fibroblasts is marked by membrane tubules that act as railways to direct cancer cell migration.

## INTRODUCTION

Cell migration is fundamental in cancer development as it is linked to tumor cell invasion and formation of metastases (*1*). Several chemical and physical cues of the tumor microenvironment were described to regulate cancer cell migration (*2*–*5*). Fibroblasts, and in particular cancer-associated fibroblasts (CAFs), act on cancer cell migration in several ways, sometimes preventing invasion by building a capsule of extracellular matrix around the tumor (*6*), and sometimes favoring cancer cells escape from the primary tumor by secreting soluble factors (*4*), modifying the extracellular matrix (*7*, *8*) or physically pulling on cancer cells (*9*). In this latter case, CAF migration directly influences cancer cell migration. Fibroblasts were shown to deposit a membranous material composed of tubules of plasma membrane emanating from retraction fibers at the cell rear during migration (*10*, *11*). More recently, coalescence of these tubules into large vesicles termed migrasomes was described in migrating fibroblasts (*12*–*14*). Migrasomes are short-lived structures that rupture soon after their formation and release their content of signaling molecules to orientate the migration of following cells (*13*). Migrasomes can also be internalized by surrounding cells and thereby transfer regulatory factors they contain to modulate recipient cells’ fate (*13*, *15*). While it is proven that fibroblasts can produce migrasomes, it is not clear whether these structures may participate in orienting cancer cell migration. In addition, depending on the cell type, migrasomes are often less numerous than the total amount of membranous tubules left behind by fibroblasts (*16*). This opens the possibility that the tubular part of the network also plays a role in directing following cells. Here, we set out to determine if and how membranous materials left on the substrate by migrating CAFs can steer cancer cell migration.

## RESULTS

**Fig. 1.**
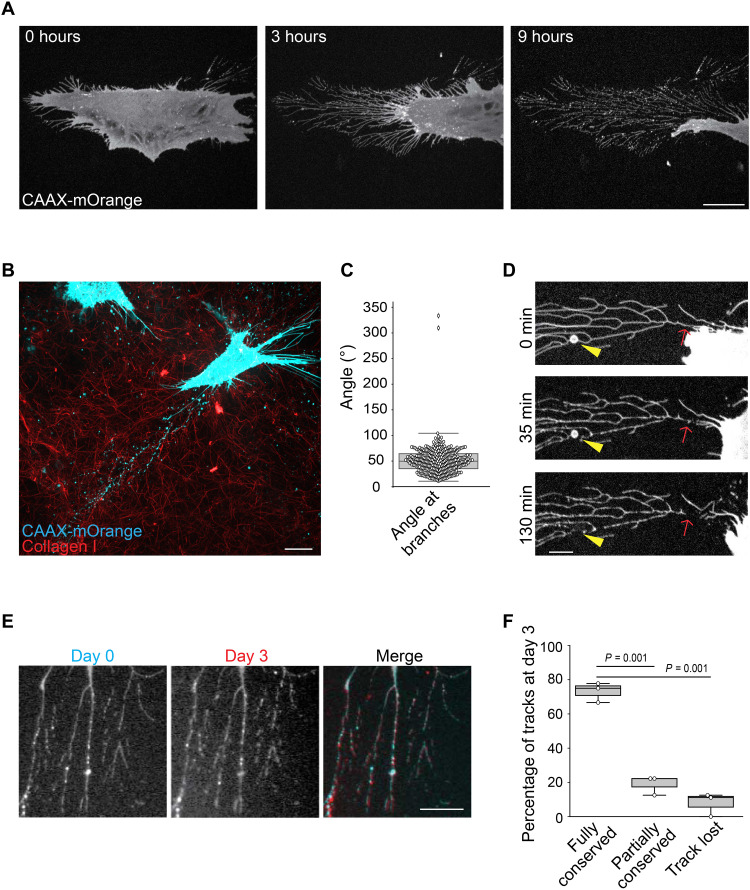
CAFs deposit membranous tracks during migration. (**A**) CAFs stably expressing CAAX-mOrange were seeded on glass and imaged by spinning disk microscopy for 9 hours. Scale bar, 20 μm. (**B**) CAAX-mOrange-expressing CAFs (blue) were embedded in a 3D network composed of collagen fibers (red) and allowed to migrate for 24 hours. Cells were then imaged by spinning disk microscopy. Scale bar, 20 μm. (**C**) Measurement of angles formed by track branches. (**D**) CAFs stably expressing CAAX-mOrange were allowed to migrate on glass and imaged by spinning disk microscopy. Arrowheads point to a migrasome and arrows indicate the site of membrane rupture. Scale bar, 5 μm. (**E**) CAFs stably expressing BFP-tagged β5-integrin were allowed to deposit tracks on glass. Tracks were imaged the first day (day 0) and then at day 3 after deposition. Scale bar, 10 μm. (**F**) Tracks as in (E) were imaged on day 0 and day 3. The histogram represents the percentage of tracks being identical between the two time points (Fully conserved), deteriorated (Partially conserved), or lost. A total of 26 tracks from three independent experiments were monitored. The percentage of each category is shown on the graph ± SD (Kruskal-Wallis test).

**Fig. 2.**
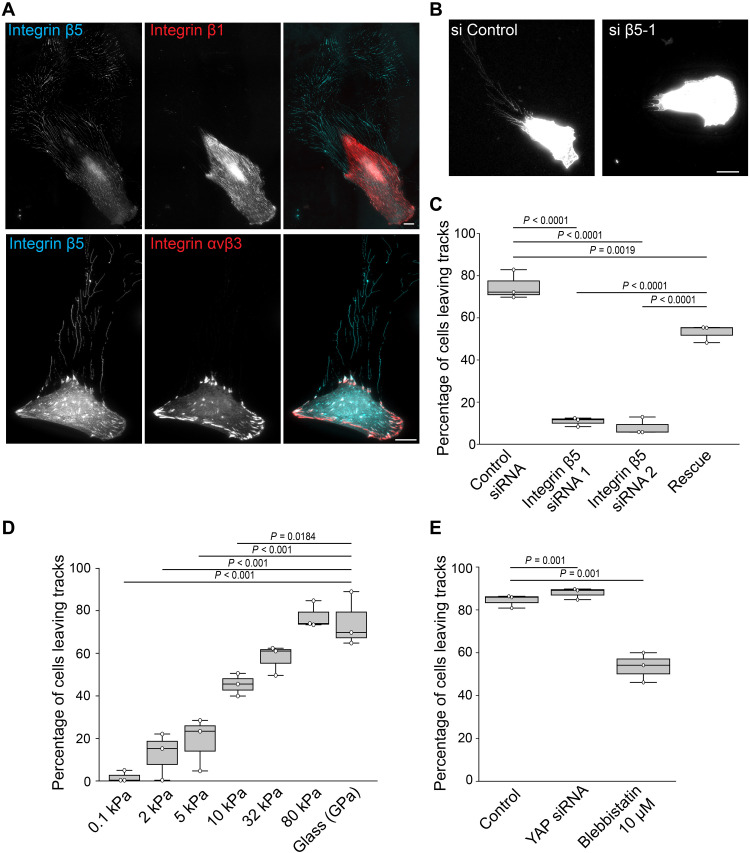
CAFs deposit tracks in a β5-integrin-dependent manner. (**A**) CAFs were allowed to migrate on glass, then fixed and stained with the indicated antibodies. Scale bars, 20 μm. (**B**) CAFs transfected with control or β5-integrin siRNA were allowed to migrate on glass for 48 hours, before cells and tracks were labeled with Alexa-488-labeleld Wheat Germ Agglutinin and imaged. Scale bar, 20 μm. (**C**) Cells were transfected with control or β5-integrin specific siRNAs and with or without a β5-encoding construct resistant to siRNA1, as indicated (condition labeled “rescue”). Cells were then monitored for track deposition. Data represent the mean percentage ± SD of cells depositing tracks from three independent experiments (Kruskal-Wallis test). (**D**) CAFs were plated on collagen-coated polyacrylamide gels of indicated rigidities and allowed to deposit tracks for 24 hours before they are fixed and stained for β5-integrin. Results are expressed as the mean percentage of cells depositing tracks ± SD from three independent experiments (one-way ANOVA, Tukey’s multiple comparison). (**E**) CAFs transfected with the indicated siRNAs were allowed to migrate on glass for 48 hours in the presence or absence of 10 μM blebbistatin. Cells were then fixed and stained for integrin αvβ5 to count how many cells would leave tracks. Data represent the mean percentage ± SD of cells depositing tracks from three independent experiments (Kruskal-Wallis test).

We observed that immortalized CAFs isolated from a colon cancer patient left behind themselves an extended network of membranous material while migrating on glass (movie S1 and Fig. 1A). The migration path of CAFs evolving in a three-dimensional (3D) network of collagen fibers was also decorated by similar membranous structures (Fig. 1B). In the 2D settings, we observed that this network originated from retraction fibers as the cell moved forward (movie S1 and Fig. 1A). Pearlation of tubules composing the network was occasionally observed, suggesting that tubules are under tension (*17*) (fig. S1A). The network was organized as branches of regular angles pointing in the direction of the CAF that left them on the substrate (Fig. 1, C and D). We also noticed some rare and large vesicles seemingly connected to the branches (Fig. 1D). However, in agreement with the literature (*12*), we observed that these vesicles, most likely corresponding to migrasomes, quickly disappeared after their formation while the branched network remained stable for days (Fig. 1, D to F). We hereafter refer to this remaining network as tracks. Interference reflection microscopy revealed that tracks deposited on glass by CAFs established very tight contacts with the substrate, suggesting that they specifically adhere to it (fig. S1B). Thus, we stained CAFs and tracks for different integrins and observed that β5-integrin but not β1- or β3-integrins accumulated in tracks (Fig. 2A). β5-integrin-positive tracks were produced by CAFs from different origins, as well as by mesenchymal stem cells and immortalized mouse osteoblasts (fig. S1C). Using β5-integrin as a specific track-marker, we then further characterized these structures. We first observed that filamentous actin and microtubules were not present in tracks (fig. S1, D and E). Then, confirming previous findings (*11*), we measured by super-resolution microscopy that tracks displayed a very regular width (fig. S1, F and G).

In agreement with the fact that β1-integrin does not localize to tracks, β1 depletion or knock-out did not prevent track formation by fibroblasts (fig. S2, A to D). Additionally, CAF migration on collagen I–coated substrates to promote β1-integrin activation also failed to produce β1-integrin-positive tracks (fig. S2E). Conversely, β5-integrin knockdown completely abrogated track biogenesis (Fig. 2, B and C, and fig. S2, F and G). The effect was not a consequence of a migration defect as β5-integrin depletion did not significantly alter CAF migration velocity (fig. S2H). Furthermore, track deposition was rescued by expressing an siRNA-resistant β5-encoding construct in β5-depleted CAFs (Fig. 2C and fig. S2I).

Integrins are well-known mechanoreceptors (*18*, *19*), and we have previously reported that β5-integrin is mechanosensitive (*20*). Hence, we hypothesized an association between track formation and substrate stiffness. Indeed, we observed that CAF deposited more tracks on stiff than on soft substrates (Fig. 2D). Depletion of the mechanoregulatory protein YAP did not affect the ability of CAFs to shed tracks on stiff (Fig. 2E and fig. S2J). On the other hand, treating CAFs with blebbistatin to inhibit myosin contractility significantly reduced track deposition (Fig. 2E). Of note, blebbistatin-treated CAFs displayed a higher migration speed as compared to control cells, indicating that migration speed and track deposition are not necessarily correlated (fig. S2H). Altogether, our results suggest that tracks are adhesive, mechanosensitive membranous networks anchored to the substrate in an integrin β5-dependent manner.

**Fig. 3.**
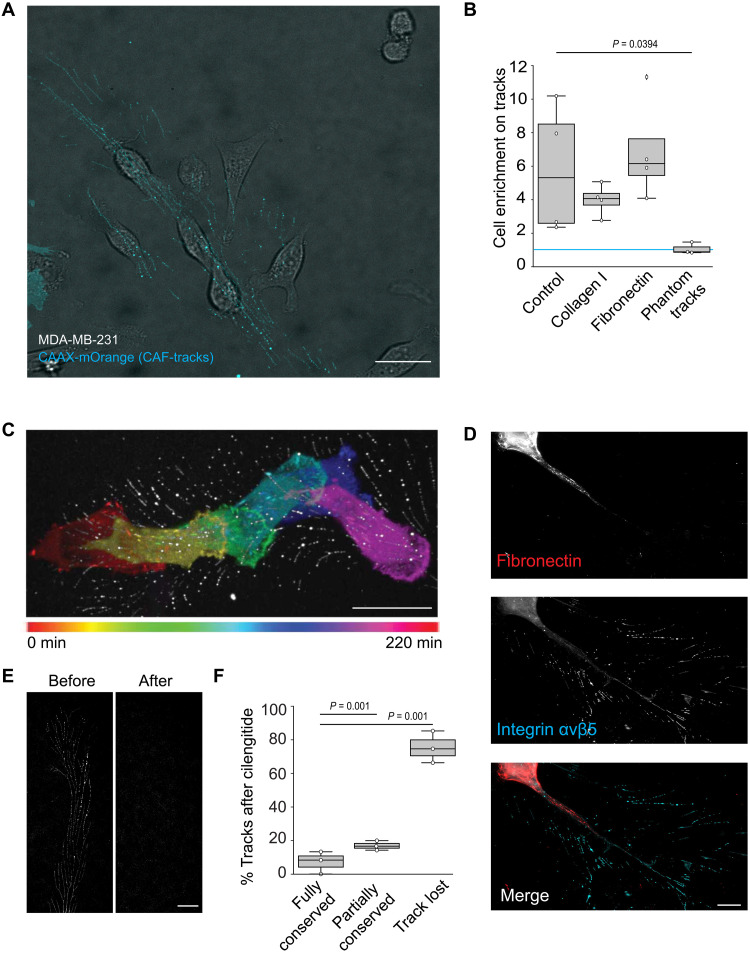
CAF-tracks represent adhesion cues for cancer cells. (**A**) MDA-MB-231 cells observed in bright field were allowed to spread on glass substrates covered with tracks deposited by CAAX-mOrange overexpressing CAFs. Scale bar, 30 μm. (**B**) β5-integrin-BFP overexpressing CAFs were allowed to deposit tracks for 48 hours on glass-bottom dishes coated or not with collagen or fibronectin, as indicated. Tracks’ positions were imaged before MDA-MB-231 cells were seeded on the substrates. For the “Phantom tracks” condition, tracks were imaged and removed from the substrate using cilengitide before seeding MDA-MB-231 cells. The number of MDA-MB-231 cells adhering in tracks areas versus in other areas of the substrates was measured. Results are expressed as the mean ratio ± SD of cell density on tracks versus in other areas of the substrate from four (three for phantom tracks) independent experiments (uncorrected Fisher’s LSD test). A value of 1 (blue line) means no enrichment on tracks. (**C**) Color-coded representation of time-lapse recording of vinculin-GFP-expressing MDA-MB-231 cells allowed to migrate for 220 min on tracks deposited by β5-integrin-BFP expressing CAFs. Scale bar, 20 μm. (**D**) CAFs were allowed to deposit tracks on glass before they are stained for αvβ5-integrin and fibronectin, as indicated. Scale bar, 10 μm. (**E**) CAFs expressing β5-integrin-BFP were allowed to migrate for 48 hours on gridded coverslips. Tracks’ positions were recorded before cilengitide was added to remove tracks and same positions were imaged again (after). Scale bar, 20 μm. (**F**) Tracks as in (E) and originating from 41 cells from three independent experiments were evaluated for their integrity after cilengitide treatment. The percentage of each category ± SD is shown (Kruskal-Wallis test).

We next investigated how cancer cells behave upon contacting CAF-tracks. First, we tested a possible role for tracks serving as adhesion cues for cancer cells. For this, CAFs were allowed to deposit tracks on the substrate for 24 hours. CAFs were then detached using a procedure that left tracks intact on the substrate (see Materials and Methods). MDA-MB-231 cancer cells were then seeded on the CAF-conditioned substrate and imaged to monitor sites of cell spreading relative to the position of tracks. MDA-MB-231 cells showed a strong preference for adhering in track regions as compared to other areas of the substrate (Fig. 3, A and B). We also noticed that upon adhering and spreading on tracks, MDA-MB-231 cells migrated by following the tracks (Fig. 3C and movie S3).

In addition to tracks, CAFs are known to deposit extracellular matrix components that may affect cancer cell adhesion (*4*, *7*). However, we noticed that fibronectin was virtually not deposited by CAFs in the low-confluency conditions used to visualize tracks (Fig. 3D). To exclude the possibility that cancer cells were sensing undetected extracellular matrix deposits, we first allowed CAFs to deposit tracks on collagen- or fibronectin-coated glass coverslips. The matrix coating slightly reduced the preferential adhesion of cancer cells to tracks’ regions although the differences were not statistically significant (Fig. 3B). This suggests that tracks represent more effective adhesion cues than collagen or fibronectin. To ensure that tracks were sufficient to support adhesion in track areas, we next recorded the position of tracks deposited by CAFs before tracks were removed using cilengitide. Indeed, cilengitide, a competitive inhibitor of αvβ5-integrin, allowed us to specifically detach tracks from the substrate (Fig. 3, E and F). We observed that MDA-MB-231 cells did not preferentially spread in regions previously occupied by tracks (Phantom tracks, Fig. 3B). This demonstrates that tracks are required and sufficient for the observed preferential adhesion in track areas. Together, our results show that tracks are very potent adhesion cues for cancer cells.

**Fig. 4.**
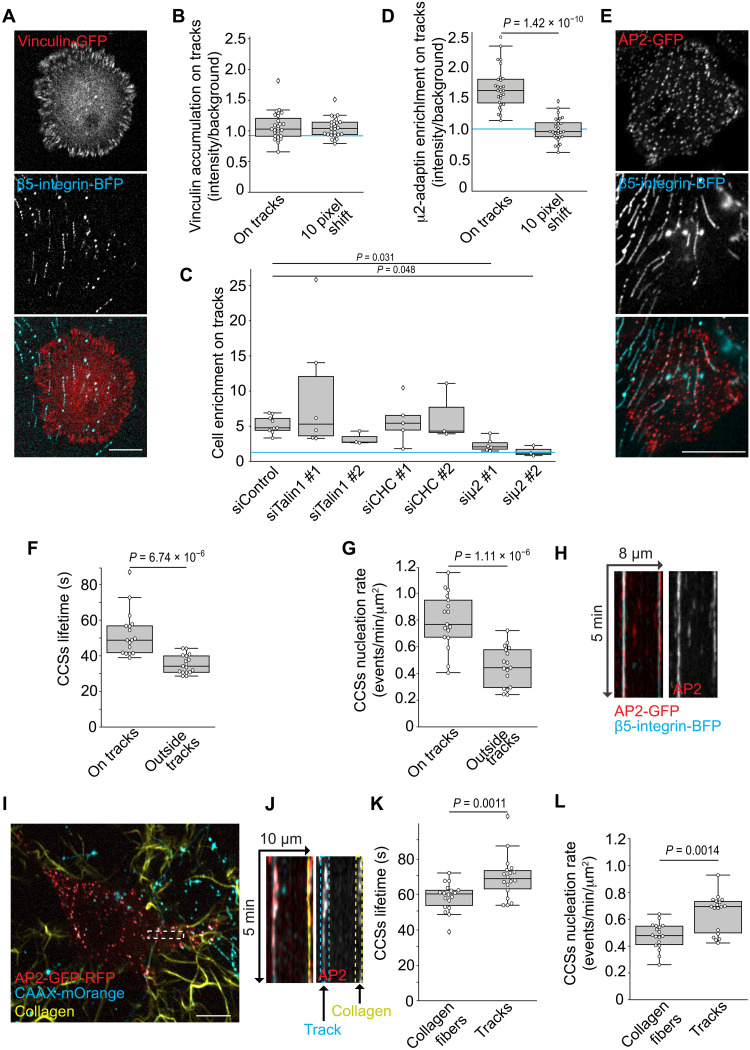
CCSs are required to adhere to CAF-tracks. (**A**) Vinculin-GFP-expressing MDA-MB-231 cells after 30 min of spreading on β5-integrin-BFP-positive tracks. Scale bar, 10 μm (**B**) Quantification of images as in (A). A total of 27 cells from three independent experiments were analyzed. A value of 1 (blue line) means no enrichment on tracks. Results are represented as mean ± SD (Student’s *t* test). (**C**) MDA-MB-231 cells transfected with the indicated siRNAs were seeded on β5-integrin-BFP-labeled tracks and imaged every 5 min for 8 hours. Results are expressed as mean ratio of cells in track versus nontrack areas ± SD from three to five independent experiments (ANOVA-Dunnett’s multiple comparison). A value of 1 (blue line) means no enrichment on tracks. (**D**) Quantification of images as in (E). A total of 28 cells from three independent experiments were analyzed. A value of 1 (blue line) means no enrichment on tracks. Results are represented as mean ± SD (Student’s *t* test). (**E**) μ2-Adaptin-mCherry MDA-MB-231 after 30 min of spreading on β5-integrin-BFP tracks. Scale bar, 10 μm. (**F** and **G**) μ2-Adaptin-mCherry MDA-MB-231 adhering on β5-integrin-BFP tracks were imaged every 5 s for 5 min. Lifetime (F) and nucleation rates (G) of CCSs on or outside tracks were measured. A total of 17 cells from three independent experiments were analyzed. Results are expressed as mean ± SD (Student’s *t* test). (**H**) Kymograph of μ2-adaptin-mCherry MDA-MB-231 adhering on β5-integrin-BFP tracks. (**I**) μ2-Adaptin-mCherry-GFP MDA-MB-231 embedded in a 3D network of fluorescent collagen fibers containing CAAX-mOrange-labeled tracks. Scale bar, 10 μm. (**J**) Kymograph of the dotted region in I. (**K** and **L**) μ2-Adaptin-mCherry-GFP MDA-MB-231 in a 3D network of fluorescent collagen fibers containing CAF-tracks were imaged every 10 s for 5 min. Lifetime (K) and nucleation rates (L) of CCSs on versus outside tracks was calculated. A total of 18 cells from three independent experiments were analyzed. Results are expressed as mean ± SD (Mann-Whitney test).

We next aimed to determine how cancer cells adhere to tracks. We first monitored the distribution of focal adhesions marked with vinculin or Talin1 in MDA-MB-231 cells adhering on tracks. Although focal adhesions were present in these cells, they never overlapped with tracks (Fig. 4, A and B, and fig. S3, A and B). Additionally, the ability of cells to preferentially adhere to tracks remained unchanged upon depletion of Talin1, an essential component of focal adhesions (Fig. 4C and fig. S3, C and D). Finally, cells depleted for Talin1 remained able to spread along tracks (fig. S3, E and F). These results suggest that focal adhesions do not regulate cancer cells adhesion on tracks. In addition, cellular contractility was not modulated by tracks as evaluated by probing for myosin light chain phosphorylation (fig. S3, G and H).

We recently showed that clathrin-coated structures (CCSs) accumulate along collagen fibers where they serve as adhesion structures in an integrin β1-dependent manner (*21*). Because tracks are tubular structures and show a similar diameter to collagen fibers (fig. S1B), we hypothesized that CCSs of cancer cells could also serve as adhesion structures to tracks. First, we observed that CCSs of MDA-MB-231 cells marked with the μ2-adaptin subunit of the AP-2 complex strongly accumulated along tracks (Fig. 4, D and E). Similar CCS accumulation was observed in HCT116 colon cancer cells adhering on tracks (fig. S4, A and B) as well as in MDA-MB-231 adhering on tracks deposited by osteoblasts, another type of fibroblastic cells (fig. S4, C and D). We observed that both CCS lifetime and nucleation rate were increased at cancer cell/track contact sites as compared to other areas of the plasma membrane (Fig. 4, F to H, and fig. S4, E to G).

This phenotype is remarkably similar to collagen fiber–pinching CCSs and suggests that CCSs could play a role in cancer cell adhesion to tracks. Indeed, CCS ablation by knockdown of the CCS core component AP-2 strongly reduced the preferential adhesion of MDA-MB-231 cells to tracks (Fig. 4C and fig. S4H). However, depletion of clathrin heavy chain (CHC) did not inhibit preferential adhesion to tracks (Fig. 4C and fig. S4I). We already reported that CHC, although required for CCS budding and endocytosis, is not required for the formation of AP-2 lattices that can serve as adhesion structures to collagen fibers (*21*, *22*). Our results suggest that CCSs on tracks, similarly to CCSs on collagen fibers, support adhesion to tracks in an endocytosis-independent manner.

We next tested whether tracks deposited by CAFs or osteoblasts differ in their capacity to influence cancer cell adhesion. CAFs and osteoblasts marked with two different plasma membrane–associated fluorophores were co-cultured and allowed to deposit tracks for 48 hours before to be removed from the substrate. We observed that CAF-tracks were slightly more potent in reinforcing cancer cell adhesion as compared to osteoblast-tracks (fig. S4J). Finally, given the similarity between tracks and collagen fibers, we decided to investigate whether tracks could bias CCS dynamics even in a more topographically complex environment. We embedded mOrange-CAAX CAFs and MDA-MB-231 cells expressing fluorescently tagged AP-2 into a 3D network of fluorescently labeled collagen I fibers to analyze CCS dynamics both along fibers and tracks. CCSs of MDA-MB-231 cells accumulated along collagen fibers and also along CAF-tracks in these 3D conditions (Fig. 4, I and J). We observed that CCSs were longer-lived and showed higher nucleation rate on tracks as compared to collagen fibers (Fig. 4, K and L). We reported that the increased lifetime of CCSs on collagen fibers reflects a state of frustrated endocytosis (*21*, *22*). Our new data suggest that CCSs located on tracks are even more frustrated.

**Fig. 5.**
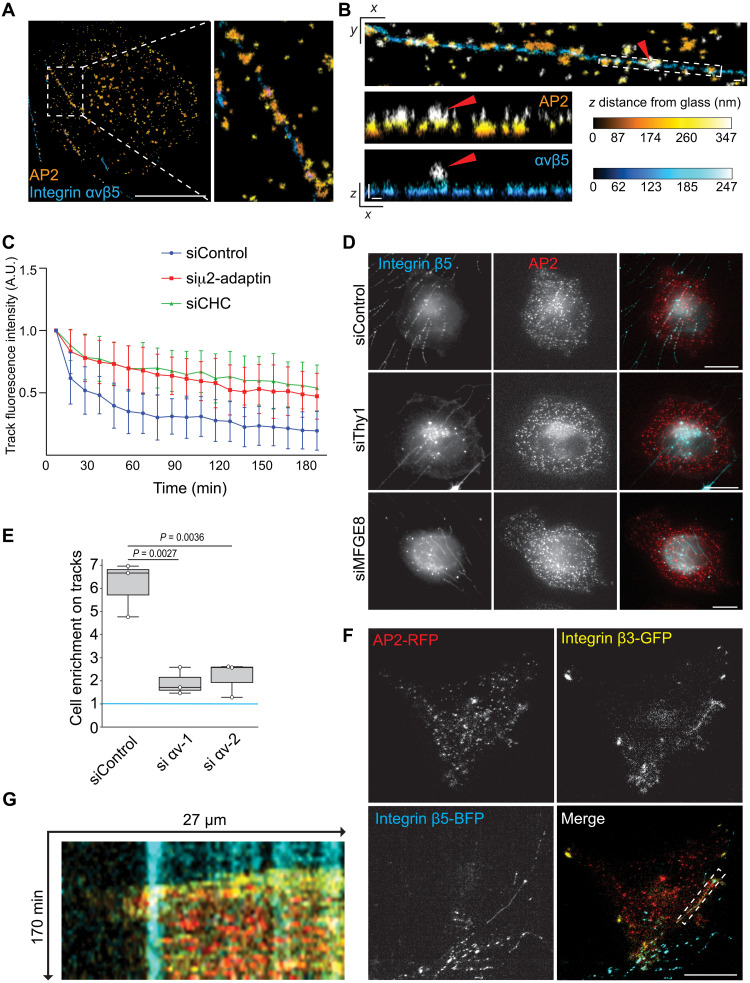
Mechanism of CCS recruitment and cancer cell adhesion on tracks. (**A**) Immunostaining of the α-adaptin subunit of AP-2 (MDA-MB-231 cells) and integrin αvβ5 (CAF-track) imaged by 3D super-resolution microscopy. Scale bar, 10 μm. (**B**) 3D super-resolution microscopy images of α-adaptin and integrin αvβ5 as in (A) and observed in *XY* and *XZ* planes, as indicated. Red arrowheads point to a budding CCS containing integrin αvβ5. *XZ* projections of the boxed area are shown. Scale bars, 200 nm. Color-coded scales indicate distance from glass in nanometers. (**C**) MDA-MB-231 transfected with the indicated siRNAs were seeded on β5-integrin-BFP tracks and imaged every 10 min for 3 hours. Data represent the evolution over time of the mean β5-integrin-BFP fluorescence intensity ± SD from three independent experiments (Dunnett’s multiple comparisons test; *P* < 0.0001 as compared to siControl). (**D**) CAFs transfected with the indicated siRNAs were allowed to migrate on glass coverslips for 48 hours to deposit tracks. MDA-MB-231 genome-edited to express μ2-adaptin-GFP-RFP were then seeded on tracks for 35 min before they are fixed and tracks were stained with β5-integrin specific antibodies. Scale bars, 10 μm. (**E**) MDA-MB-231 transfected with the indicated siRNAs were seeded on β5-integrin-BFP tracks and imaged every 5 min for 8 hours. The number of cancer cells adhering on tracks versus outside of tracks was quantified and expressed as a mean ratio ± SD from three independent experiments (Kruskal-Wallis test). A value of 1 (blue line) means no enrichment on tracks. (**F**) MDA-MB-231 genome-edited to express μ2-adaptin-mCherry and stably expressing β3-integrin-GFP were seeded on β5-integrin-BFP osteoblast-tracks and imaged by TIRF microscopy every 5 min for 8 hours. Representative images of the initial phase of MDA-MB-231 cells spreading on tracks are shown. Scale bar, 20 μm. (**G**) Kymograph corresponding to the boxed area in (F).

We next observed by super-resolution microscopy that CCSs wrapped around and pinched tracks labeled with β5-integrin (Fig. 5A and movie S4). We also noticed that some CCSs forming on tracks showed a budded profile and were filled with β5-integrin (Fig. 5B), suggesting that CCSs can internalize portions of tracks. Indeed, we observed that tracks progressively disappeared below MDA-MB-231 cells adhering on them and this was dependent on both AP-2 and clathrin expression (Fig. 5C). Our results suggest that CCSs on tracks experience a transient period of frustration, as reflected by their longer lifetime, but eventually manage to internalize portions of tracks. Actin has been proposed to be recruited at frustrated CCSs that experience difficulties to bud because of mechanical constraints (*23*–*27*). Indeed, we observed that inhibiting actin dynamics with Cytochalasin D resulted in an increased accumulation of stable CCSs along tracks (fig. S5, A and B). In addition, Cytochalasin D incubation prevented tracks’ disappearance below MDA-MB-231 cells (fig. S5, A to C, and movie S5). Thus, our results suggest that actin dynamics assists CCS budding on tracks and allows track uptake.

We next decided to investigate how CCSs are recruited on tracks. We reasoned that some CCS cargos may engage proteins expressed at the surface of tracks. We thus performed mass spectrometry analyses of purified tracks (see Materials and Methods) to identify potential candidates. First, we observed that the fraction enriched for tracks contained β5-integrin and its partner integrin αv (table S1), which was then confirmed by Western blot analyses (fig. S5D). Among the identified hits, we focused on plasma membrane–associated proteins involved in cell-cell adhesion. CAFs depleted for these proteins could still deposit tracks, so we seeded cancer cells on these tracks and measured CCS accumulation on tracks. We observed a reduced accumulation of CCSs along tracks depleted of Thy1 or MFGE8 proteins (Fig. 5D and fig. S5, E and F). Importantly, we confirmed the presence of these two proteins in tracks by immunofluorescence (fig. S5G). Both Thy1 and MFGE8 were reported to bind in trans to the αvβ3 integrin dimer (*28*–*30*). We first confirmed that both MDA-MB-231 and HCT116 expressed αv- and β3-integrins (fig. S6A). We next observed that integrin αv knockdown reduced the preferential adhesion of MDA-MB-231 cells on wild-type tracks (Fig. 5E and fig. S6B). In addition, we observed that GFP-tagged integrin β3 strongly but transiently accumulated along tracks during cancer cell spreading (movie S6). Occasional colocalization between CCSs and GFP-β3 was observed during MDA-MB-231 cells spreading on tracks (Fig. 5, F and G, and movie S6). In addition, inhibiting track uptake with Cytochalasin D led to an increased and sustained accumulation of GFP-β3 along tracks (fig. S6, C and D). These latter observations suggest that αvβ3 transiently accumulates along tracks before it is removed through endocytosis, most likely together with portions of tracks. The adaptor proteins DAB2 and Numb are reported to bridge integrin αvβ3 to the clathrin endocytic machinery (*31*). We observed that DAB2 depletion, but not Numb depletion, led to a reduction of CCS recruitment along CAF-tracks (fig. S6, E and F). Overall, our results show that αvβ3 in MDA-MB-231 cells and αvβ3-interacting receptors on tracks are required for CCS-dependent cancer cell adhesion to tracks.

**Fig. 6.**
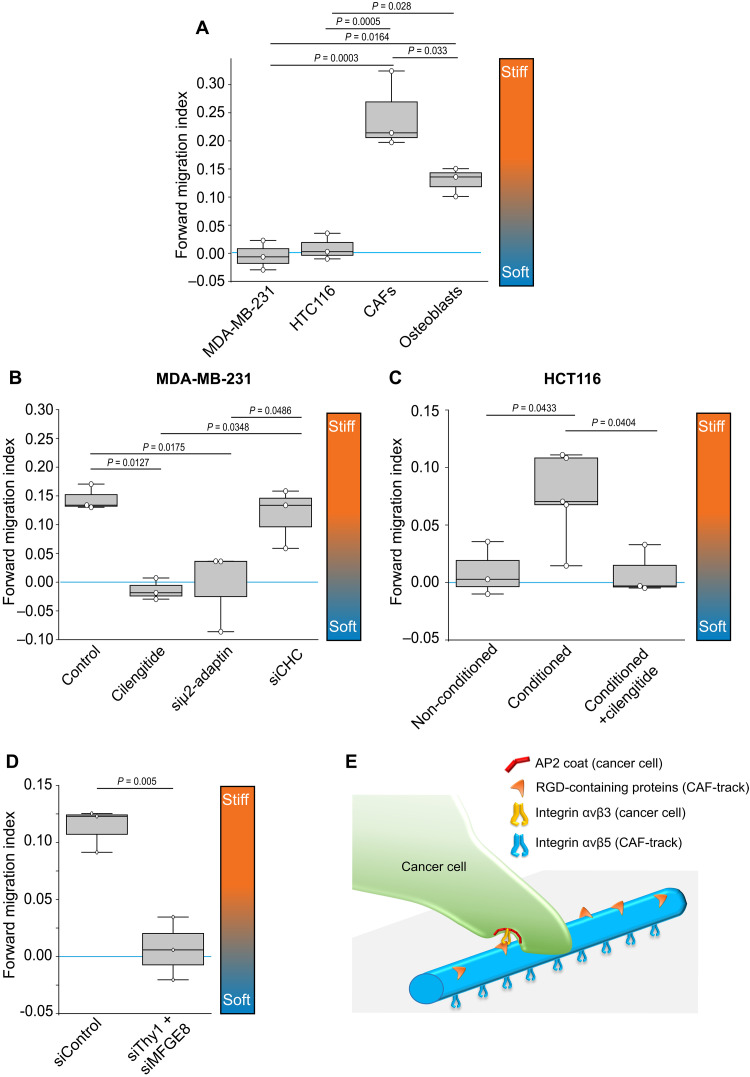
CAF-tracks orientate cancer cell migration. (**A**) Cells seeded on collagen-coated rigidity gradients were imaged every 20 min for 14 hours, manually tracked, and forward migration index (FMI) was calculated using the Chemotaxis tool FIJI plugin. Values close to 0 (blue line) indicate random migration. Gradient orientation is shown. A total of 350 MDA-MB-231, 204 CAFs, 514 HTC116, and 505 osteoblasts were tracked in three independent experiments. Data are expressed as mean FMI ± SD (ANOVA-Tukey’s multiple comparison). (**B**) Control or siRNA-treated MDA-MB-231 cells were seeded on collagen-coated rigidity gradients conditioned by CAFs and treated or not with cilengitide. Cells were imaged every 20 min for 14 hours and manually tracked, and FMI was calculated using the Chemotaxis tool FIJI plugin. Values close to 0 (blue line) indicate random migration. Gradient orientation is shown. A total of 281 (control), 626 (cilengitide), 424 (siμ2-adaptin), and 362 (siCHC) cells were tracked from three independent experiments. Data are expressed as mean FMI ± SD (ANOVA-Tukey’s multiple comparison). (**C**) HCT116 cells were seeded on collagen-coated rigidity gradients conditioned or not by CAFs and treated or not with cilengitide. Cells were imaged every 20 min for 14 hours and manually tracked, and FMI was calculated using the Chemotaxis tool FIJI plugin. Values close to 0 (blue line) indicate random migration. Gradient orientation is shown. A total of 514 (nonconditioned), 1039 (conditioned), and 465 (conditioned + cilengitide) cells were tracked from three to five independent experiments. Data are expressed as mean FMI ± SD (ANOVA-Dunnett’s multiple comparison). (**D**) MDA-MB-231 cells were seeded on collagen-coated rigidity gradients conditioned by CAFs, imaged every 20 min for 14 hours, and manually tracked, and FMI was calculated using the Chemotaxis tool FIJI plugin. Values close to 0 (blue line) indicate random migration. Gradient orientation is shown. A total of 736 (siControl) and 877 (siThy1 + siMFGE8) cells from three independent experiments were tracked. (**E**) Model of cancer cell adhesion along CAF-tracks.

Our findings suggest that tracks represent adhesion cues for cancer cells. We reasoned that such adhesion cues may also orient cancer cell migration. Fibroblasts perform durotaxis when seeded on substrates presenting nonhomogeneous rigidities (*32*–*34*). We first confirmed that CAFs efficiently migrated toward the stiffer end of a rigidity gradient (Fig. 6A and fig. S6, A and B). However, MDA-MB-231 and HCT116 cells did not durotax on this steep rigidity gradient, but rather migrated randomly (Fig. 6A). Because CAFs deposit more tracks on stiff than on soft substrates (Fig. 2D), we reasoned that the asymmetric distribution of tracks on a rigidity gradient could function as a haptotactic cue to orient cancer cell migration. To test this hypothesis, CAFs were allowed to migrate and deposit track on the rigidity gradient before they are removed. We then seeded cancer cells on the CAF-conditioned rigidity gradient and monitored their migration. We observed that both MDA-MB-231 and HCT116 migrated toward the stiffer end of the CAF-conditioned gradient (Fig. 6, B and C). Similar results were obtained when MDA-MB-231 cells migrated on an osteoblast-conditioned rigidity gradient (fig. S6C and Fig. 6A). However, cilengitide-mediated track removal before cancer cell seeding resulted in both cell lines migrating randomly (Fig. 6, B and C). Thus, we concluded that tracks deposited by CAFs regulate the direction of cancer cell migration. We next tested the role of CCSs in this directed migration assay. We observed that AP-2 subunit μ2-adaptin knockdown prevented MDA-MB-231 cells to drift toward the more rigid side of the CAF-conditioned gradient (Fig. 6B). However, in line with the nonrequirement for clathrin in mediating adhesion to tracks (Fig. 3C), CHC-depleted cells still migrated to the stiffer end of the CAF-conditioned gradient (Fig. 6B). Finally, we depleted Thy1 and MFGE8 from CAFs and we tested whether tracks lacking these two proteins could still orient cancer cell migration. MDA-MB-231 migrated randomly in these conditions (Fig. 6D). Collectively, our results show that tracks guide the migration of cancer cells and that this is regulated by CCS-mediated adhesion to tracks.

## DISCUSSION

Overall, our results show that migrating fibroblasts deposit membranous material in the form of tracks and that cancer cells can use these tracks to steer their migration. We provide evidence that β5-integrin is required for fibroblast-track generation. Previous work on β5-integrin highlighted the unique characteristic of such receptor to localize to both focal adhesions and reticular adhesions (*20*, *35*, *36*). It is hence possible that tracks emanate from the two different types of adhesion structures and it would be important to address this point in the future. CCSs play an instrumental role in cancer cell migration along tracks, as they are required to adhere to tracks (Fig. 6E). Collagen fibers and tracks share common traits such as their tubular shape and diameter. The difference, however, is that CCSs cannot internalize collagen fibers because these structures are too long and too rigid to be accommodated into clathrin-coated pits (*21*). Nevertheless, in the case of tracks, which are membranous structures and are thus less rigid than collagen fibers, CCSs can internalize portions of them with the necessary assistance of actin dynamics. Thus, tracks may represent an intercellular communication system that is read and erased by CCSs of following cancer cells. In addition to the tracks described here, migrasomes have been described to give rise to local chemotactic gradients driving cell migration during zebrafish gastrulation in vivo (*13*). However, we did not identify any of the described migrasome-specific chemo-attractants (*13*) in our mass spectrometry analysis of tracks, further suggesting that migrasomes are unlikely to play an important role in the mechanisms we report here.

Deposition of secreted extracellular vesicles by cancer cells has been proposed to orient the migration of following cells, acting as chemotactic signals (*37*). Also, paths made of fibronectin deposited by migrating cells were shown to represent self-induced environmental perturbations that influence future cell trajectories (*38*). Thus, migrating cells may assemble different types of paths that influence self and/or neighboring cell migration. Here, we propose that tracks encode a long-lived but erasable spatial memory of CAF migration trajectories that instructs cancer cell migration. Because tracks are observed in many different cell types, these extracellular structures may influence many different migration-dependent processes during development, homeostasis, and diseases.

## MATERIALS AND METHODS

### Cell lines and constructs

hTert-immortalized CAFs stably expressing mOrange-CAAX (a gift from D. Matic Vignjevic, Insitut Curie, Paris, France) and desmoplastic small round cell tumor CAFs (a gift from C. Hénon, Gustave Roussy) were grown in Dulbecco’s modified Eagle’s medium (DMEM) Glutamax supplemented with 10% fetal calf serum (FCS) and 1% insulin-transferrin-selenium (ITS ref. I3146, Sigma-Aldrich) at 37°C in 5% CO_2_. Wild type and β1-integrin Knock Out immortalized mouse osteoblasts (a gift from C. Albiges-Rizo, IAB Grenoble, France), MDA-MB-231 cells (a gift from P. Chavrier, Institut Curie, Paris, France) and genome-edited MDA-MB-231 cells engineered to express an endogenous GFP- or mCherry-tagged μ2 subunit (a gift from D. Drubin, University of California-Berkeley, California, USA), HEK293T (a gift from P. Chavrier, Institut Curie, Paris, France), and HCT116 (a gift from D. Matic Vignjevic, Institut Curie, Paris, France) were grown in DMEM Glutamax supplemented with 10% FCS at 37°C in 5% CO_2_. All cell lines were periodically tested for the absence of mycoplasma contamination. All experiments were performed on hTert-immortalized CAFs stably expressing mOrange-CAAX, unless otherwise stated.

Substrates were coated with collagen I (50 μg/ml; Thermo Fisher Scientific, ref. A10483-01) or fibronectin (20 μg/ml; Corning, ref. 354008), or left uncoated. Plasmids encoding for β5-integrin were described previously (*20*). Briefly, polymerase chain reaction–purified β5-integrin with engineered flanking restriction sites (Xho I/Bam HI) was subcloned into the multi-cloning sites of pEGFP-N1 (Clontech) to encode an in-frame fusion protein with the C-terminal EGFP-tag (pEGFP-N1-β5-integrin) or with C-terminal mCherry-tag (mCherry-N1-β5-integrin). SiRNA-resistant β5-integrin was obtained by site-directed mutagenesis of the β5-encoding cDNA at the following positions: A69T, T72G, C73G, and C75G (silent mutations—resistant to siβ5-1). pHR GFP-CAAX (for lentiviral production) was a gift from R. Vale (Addgene plasmid #113020).

Lentiviral plasmids encoding for integrin β3-GFP, integrin β1-GFP [built as in Huet-Calderwood *et al.* (*39*)], integrin αv-mCherry, β5-integrin-BFP, and Vinculin-GFP were purchased from VectorBuilder and are available upon request. Lentiviruses were produced in HEK293T cells using the packaging plasmid psPAX2 and the VSV-G envelope expressing plasmid pMD2.G according to the Trono lab protocol. To generate stable cell lines, cells were transduced with lentiviruses and sorted via fluorescence-activated cell sorting.

Plasmids were transfected 24 hours after cell plating using either Lipofectamine 3000 according to the manufacturer’s instructions (MDA-MB-231) or by electroporating cells in suspension using AMAXA nucleofector Kit V program A-024 (CAFs) or program X-001 (osteoblasts) according to the manufacturer’s instructions. Alternatively, linear Polyethylenimine (PEI) (molecular weight, 25,000; Polysciences catalog no. 23966) at 1 mg/ml was used to transfect 50% confluent cells in a six-well plate according to the following protocol: 2 μg of DNA was added to 100 μl of OptiMEM, followed by the addition of 4 μl of PEI, vortex, and incubation for 10 min at room temperature (RT) before adding the mix to the cells.

### Antibodies and drugs

Rabbit polyclonal anti-α-adaptin antibody (ref. sc-10761), mouse monoclonal anti-YAP (ref. sc101199), mouse monoclonal anti-Thy1 (ref. sc-53456), mouse monoclonal anti-MFGE8 (ref. sc-8029), and mouse monoclonal anti–integrin β1 (ref. sc-59827) were purchased from Santa Cruz Biotechnology Inc. (Santa Cruz, CA, USA). Mouse monoclonal anti–α-adaptin antibody (ref. ab 2807), rabbit monoclonal anti-AP2M1 (ref. 75995), rabbit polyclonal anti-Talin1 (ref. ab71333), and mouse monoclonal anti–integrin αvβ3 (ref. ab190147) were purchased from Abcam. Mouse monoclonal anti-CHC (ref. 610500) antibody and mouse monoclonal anti–integrin αv (ref. 611012) were obtained from BD Transduction Laboratories (Becton Dickinson France SAS, Le Pont-De-Claix, France). Mouse monoclonal anti–α-tubulin (ref. T6199) was purchased from Merck. Rabbit anti–integrin β1 was a gift from the Albiges-Rizo’s lab. Rabbit monoclonal anti–β5-integrin (ref. 3629) was purchased from Cell Signaling Technology. Mouse monoclonal anti–integrin αvβ5 (ref. MAB1961) and anti–integrin β3 (ref. AB2984) were purchased from Millipore. Rabbit polyclonal anti-Thy1 (ref. A2126) and rabbit polyclonal anti-MFGE8 (A12322) were used for Western blot and purchased from ABclonal. HRP-conjugated anti-mouse (ref. 115-035-062) for Western blot was from Jackson ImmunoResearch Laboratories (West Grove, PA, USA). HRP-conjugated anti-rabbit (ref. A0545) was purchased from Sigma-Aldrich.

Alexa-conjugated secondary antibodies anti-mouse-A488 (ref. A21208), anti-mouse-A647 (ref. A31571), anti-rabbit-A647 (ref. A31573), and anti-rabbit-A488 (ref. A21206) were from Molecular Probes (Invitrogen). Alexa-conjugated antibodies A568 (ref. ab175471) were obtained from Abcam. Anti-mouse CF633 (ref. 20124) was from Biotium. Phalloidin A488 (ref. A 12379), Phalloidin-A633 (ref. A22284), WGA-A633 (ref. W-21404), and WGA-A488 (ref. W-11261) were from Molecular Probes (Invitrogen). For Stochastic Optical Reconstruction Microscopy (STORM) microscopy, CF647 F(ab′)2 (ref. BTM20042) and CF680 F(ab′)2 (ref. BTM20064) were bought from Ozyme.

Rat tail collagen I (ref. A10483-01) and Fibronectin (Corning, 354008) were purchased from GIBCO. Green Fluorescent HiLyte 488 Fibronectin (ref. FNR02-A) was purchased from Cytoskeleton and used at 20 μg/ml. Cytochalasin D (ref. 22144-77-0) and Blebbistatin (ref. B0560) were purchased from Sigma-Aldrich and used at a final concentration of 10 μM. Cilengitide was purchased from Selleckchem (catalog no. S7077) and used at a final concentration of 10 μM. Calyculin A (ref. PHZ1044) and Calcein AM (ref. 65-0853-39) were purchased from Thermo Fisher Scientific and used at a final concentration of 20 nM and 1 μM, respectively. For Western blot experiments, cells were lysed in ice-cold MAPK buffer (100 mM NaCl, 10 nM EDTA, 1% IGEPAL CA-630, 0.1% SDS, and 50 mM Tris-HCl, pH 7.4) supplemented with protease and PhosSTOP phosphatase inhibitor (ref. 04 906 845 001, Roche). Antibodies were diluted at 1:1000 in phosphate-buffered saline (PBS)–0.1% Tween–5% nonfat dried milk.

### RNA interference

For siRNA-mediated depletion, 150,000 cells were plated together with the indicated siRNA (30 nM) using RNAimax (Invitrogen, Carlsbad, CA) according to the manufacturer’s instruction. Protein depletion was checked after 72 hours of siRNA treatment by Western blot or immunofluorescence with specific antibodies. To perform depletions of AP2, CHC, and Talin1 in MDA-MB-231, as well as to perform depletions in CAFs, cells were transfected once as described above and then a second time, 48 hours later, with the same siRNAs. In this case, cells were analyzed 96 hours after the first transfection. The sequences of the siRNAs used are reported in table S2.

### Indirect immunofluorescence microscopy and fluorescence quantification

Cells were fixed in ice-cold methanol or 4% Paraformaldehyde (PFA) and processed for immunofluorescence microscopy by using the indicated antibodies. For anti-Talin1 staining, cells were briefly pre-extracted for 30 s using PEM buffer (PIPES 80 mM, EGTA 5 mM, MgCl_2_ 2 mM, pH 6.8, and 1% Triton X-100) before PFA fixation. Coverslips were mounted with Fluoromount-G (ref. 0100-20–ref. 0100-01 SouthernBiotech).

Immunofluorescence images were acquired on a Leica Thunder epifluorescence microscope (Leica Microsystems Ltd., Wetzlar, Germany) through 63× [Numerical Aperture (N.A.) 1.40], 20× (HC N.A. 0.40 PH1), 10× (Fluotar N.A. 0.32 PH1), or 5× (PLAN 5/N.A. 0.12 PHO) objectives. The microscope was equipped with an LED5 lamp from Leica, a filter cube DFT51010, an external wheel EFW for DMi8, and an sCMOS K5 camera. A PeCon chamber i8 was installed on the microscope to perform live imaging at 37°C with 5% CO_2_. The microscope was steered by the Leica dedicated LasX software with Navigator, allowing the generation of mosaic stitched images.

To quantify track deposition, transfected CAFs were plated at a density of 40–50 cells/mm^2^ and allowed to migrate for 24 to 48 hours. Random regions between 2 and 6 mm^2^ (enough to capture at least 100 cells) were imaged and cells leaving tracks were counted.

To monitor the stability of tracks over time, cells were plated on 35 mm glass-bottom dishes with grids of 50 μm (ref. P35G-1.5–14-C-GRID - MatTek Corporation). Images were acquired 2 days after cell plating. With the help of the grids, the same regions were imaged 72 hours later and the number of tracks still present on the surface was quantified.

To quantify CCSs and FA enrichment along tracks, 12,000 CAFs were plated on 18 mm–diameter coverslips for 48 hours, then 60,000 genome-edited MDA-MB-231 cells wt were engineered to express an endogenous GFP-tagged μ2 subunit, or stably expressing vinculin-GFP was added for 35 min. Cells were then fixed with PFA and β5-integrin was stained to visualize tracks. Alternatively, staining against β5-integrin and α-adaptin subunit of AP-2 or Talin1 was performed. Tracks under MDA-MB-231 were manually segmented and the fluorescence intensity of CCSs or FAs markers in the region corresponding to tracks was quantified. The masks were then shifted by 10 pixels to calculate the background, nonspecific GFP average fluorescence intensity. The ratio between the GFP signal under the tracks versus the GFP signal in the shifted region was used to quantify enrichment of CCSs or FA along tracks. All quantifications on immunofluorescence images were done with FIJI after background subtraction.

### Acrylamide gels of controlled stiffness

Coverslips or glass-bottom dishes (ibidi, ref. 81218-200) were incubated with APTMS (3-aminopropyltrimethoxysilane) for 15 min at RT, then washed extensively with water, and incubated for 30 min with glutaraldehyde 0.5% in PBS and washed again with water. Acrylamide 40% and bis-acrylamide 2% were mixed (5% and 0.04% for 0.1 kPa gels, 7.5% and 0.06% for 5 kPa gels, 18% and 0.4% for 31.7 kPa gels, and 16% and 0.96% for 80 kPa gels, respectively) with PBS, APS 10%, and tetramethylethylenediamine (TEMED). Nine microliters of this solution was polymerized on the treated glass. Gels were washed with PBS, followed by a 30 min incubation with 300 μl of 50 mM Hepes, pH 7.5, + 100 μl of sulfo-SANPAH (1 mg/ml in 50 mM Hepes pH 7.5) + 100 μl EDC (10 mg/ml in 50 mM Hepes, pH 7.5). Gels were subsequently cross-linked under ultraviolet (UV) light for 10 min, washed, and incubated with collagen I (50 μg/ml) overnight at 37°C. The elasticity of the different gels was controlled by atomic force microscopy (AFM) as indicated in (*40*) The generation of 80 kPa gels was performed according to a previously published protocol (*41*). Durotaxis gradients were obtained by placing 4 μl of 0.1 kPa solution next to 4 μl of 80 kPa solution on a treated glass-bottom dish. Fluorescent beads of 500-nm diameter (ref. F8812, Invitrogen) were added to one of the two solutions to visualize the gradient. An 18 mm–diameter glass coverslip was then quickly placed on top of the droplets, thus allowing for mixing of the two solutions and generation of a rigidity gradient. This method generates rigidity gradients where the stiffness increases linearly for 1 to 2 mm, as verified by AFM. Durotaxis gradients were coated with collagen I (50 μg/ml) overnight at 37°C. Imaging was started 5 hours after cell plating and performed at the center of the gradient, which was determined by looking at the fluorescent beads inside the gels. The number of cells plated on a gradient was 50,000 for CAFs and 100,000 for the other cell lines. One fluorescence image was acquired to visualize the fluorescent beads and identify the center of the gradient. Then, videos of 12 to 14 hours were recorded at a frequency of one image every 20 min with phase contrast. Cells were manually tracked using the “Manual tracking” plugin of FIJI. Forward migration indexes were obtained with the Ibidi “Chemotaxis and Migration Tool” in FIJI.

### Live cell spinning disk microscopy and quantifications

Cells were imaged on a Nikon Ti2 Eclipse (Nikon France SAS, Champigny sur Marne, France) inverted microscope equipped with a 60× NA 1.40 oil objective WD 0.130, an sCMOS PRIME 95B camera (Photometrics, AZ, USA), and a dual-output laser launch, which included 405, 488, 561, and 642 nm 30 mW lasers. The emission filter characteristics are as follows: 452/45 nm (Semrock Part# FF01-452/45); 470/24 nm (Chroma 348716); 525/50 nm (Semrock Part# FF03-525/50); 545/40 nm (Chroma 346986); 6986); (Semrock Part# FF01-609/54); and 708/75 nm (Semrock Part# FF01 708/75). The microscope was steered by Metamorph 7 software (MDS Analytical Technologies, Sunnyvale, CA, USA).

For CCS dynamics quantification along tracks, cells were imaged at one image every 5 s for 5 min for 2D experiments and one stack (2 to 3 μm) every 10 s for 5 min for 3D experiments. Tracks were manually segmented and CCS lifetime was measured on tracks versus outside tracks using the TrackMate plugin of FIJI (2D experiments) or manually (3D experiments). Tracks below 5 s of duration (detected on only one frame) were discarded. To quantify CCS nucleation, the number of CCSs appearing during the 5 min long video was counted and normalized to the area. At least 1000 CCS-tracks from at least five cells per condition and per experiment were quantified in three to five independent experiments. Data are expressed as mean ± SD. Videos of 8 to 12 hours were recorded at a frequency of one image every 5 min. When needed, before image analysis, videos were realigned either manually or using the Template Matching plugin of FIJI.

For experiments concerning adhesion along tracks, 1000 CAFs or osteoblasts (30 cells/mm^2^) were plated in a culture-insert 4-well (ibidi, ref. 80466) and allowed to migrate for 24 to 48 hours. More than five regions containing tracks were then randomly selected in each well and 3000 cancer cells were added in each well. Cell adhesion and migration were monitored for 8 hours and the number of cancer cells spreading on tracks was counted. Results were then normalized by the area covered by tracks. Fluorescent fibronectin and collagen were used to visually verify homogeneous extracellular matrix coating.

### TIRF microscopy

For total internal reflection fluorescent microscopy (TIRF), MDA-MB-231 cells transfected with the indicated plasmids were imaged through a 100× 1.49 NA APO TIRF WD 0.13 to 0.20 oil objective lens on a Nikon Ti2 Eclipse (Nikon France SAS, Champigny sur Marne, France) inverted microscope equipped with an sCMOS PRIME 95B camera (Photometrics, AZ, USA) and a dual-output laser launch, which included 405, 488, 561, and 642 nm 30 mW lasers, and driven by Metamorph 7 software (MDS Analytical Technologies, Sunnyvale, CA, USA). A motorized device (Piezo electrical XYZ stage from Nikon) driven by Metamorph allowed the accurate positioning of the illumination light for evanescent wave excitation. Videos of 8 to 12 hours were recorded at a frequency of one image every 5 min. When needed, before image analysis, videos were realigned either manually or using the Template Matching plugin of FIJI.

### Interference reflection microscopy

Images were acquired on a Leica Sp8 confocal microscope with a Pecon incubation chamber equipped with two hybrid and three PMT detectors, using 405, 488, 561, and 633 nm lasers and an 85/15 cube. Imaging was performed using a 63× objective (1.4 NA) and the 633 nm laser was used to have the IRM picture. Fast Fourier transforms (FFTs) of the raw images were obtained in FIJI and used to remove linear interferences. Final images were obtained using the inverse FFT command on FIJI.

### 3D migration in collagen networks

For 3D cell migration assays, a glass-bottom dish was treated with poly-l-lysine 0.1% (Sigma-Aldrich, ref. P8920) for 5 min at RT before adding a 50 μl droplet of collagen type I (BD Biosciences, 50:1 ratio unlabeled versus Alexa548-labeled) at a final concentration of 2.2 mg/ml mixed with 5000 CAFs CAAX-mOrange cells. Collagen was allowed to polymerize at room temperature for 30 min before immerging the setup in pre-warmed DMEM supplemented with FCS and ITS. Twenty-four hours later, cells were imaged by spinning disk microscopy.

### Proteomic analysis of CAF-tracks

CAFs were plated at a density of 40 cells/mm^2^ in six T75 cell culture flasks. After 48 hours of migration, DMEM + ITS + FCS was replaced with DMEM + ITS. Twenty-four hours later, Calyculin A was added (final concentration, 20 nM) for 20 min to induce cell detachment from the substrate. Flasks were then washed five times with PBS. DMEM (3 ml) was added to each flask. Cilengitide, a selective inhibitor of the integrins αvβ3 and αvβ5, was added to three flasks to harvest tracks (final concentration, 20 μM), while dimethyl sulfoxide was added to the other three flasks. After 80 min incubation at 37°C, the medium was recovered and centrifuged at 20,000*g* for 30 min. Pellets were then recovered in 20 μl of PBS and subjected to further analysis.

Each sample was dried and solubilized in 10 μl of 8 M urea and 200 mM ammonium bicarbonate and then reduced in 5 mM dithiothreitol, pH 8, with vortexing at 57°C for 30 min. After cooling to room temperature, cysteines were alkylated by adding 10 mM of iodoacetamide for 30 min in the dark. After diluting to 1 M urea with 200 mM ammonium bicarbonate pH 8.0, samples were digested with 0.4 μg trypsine/LysC (Promega) overnight, with vortexing at 37°C. Samples were then loaded onto homemade C18 StageTips for desalting. Peptides were eluted using 40/60 MeCN/H2O + 0.1% formic acid, vacuum concentrated to dryness, and reconstituted in 10 μl or 20 μl injection buffer (0.3% TFA) before nano-liquid chromatography-tandem mass spectrometry (nano-LC-MS/MS) analysis. Online chromatography was performed using an RSLCnano system (Ultimate 3000, Thermo Fisher Scientific) coupled to an Orbitrap Fusion Tribrid mass spectrometer (Thermo Fisher Scientific). Peptides were trapped on a C18 column (75 μm inner diameter × 2 cm; nanoViper Acclaim PepMapTM 100, Thermo Fisher Scientific) with buffer A (2:98 MeCN:H_2_O in 0.1% formic acid) at a flow rate of 3.0 μl/min over 4 min. Separation was performed on a 50 cm by 75 μm C18 column (nanoViper Acclaim PepMapTM RSLC, 2 μm, 100 Å, Thermo Scientific), regulated to a temperature of 40°C with a linear gradient of 3% to 32% buffer B (100% MeCN in 0.1% formic acid) at a flow rate of 150 nl/min over 91 min. Full-scan MS was acquired using an Orbitrap Analyzer with the resolution set to 120,000, and ions from each full scan were higher-energy C-trap dissociation (HCD) fragmented and analyzed in the linear ion trap. For identification, the data were searched against the *Homo sapiens* UP000005640 database using Sequest HT through Proteome Discoverer (v.2.4). Enzyme specificity was set to trypsin and a maximum of two miss cleavages sites were allowed. Oxidized methionine, Met-loss, Met-loss-Acetyl, and N-terminal acetylation were set as variable modifications. Carbamidomethylation of cysteins were set as fixed modification. Maximum allowed mass deviation was set to 10 ppm for monoisotopic precursor ions and 0.6 Da for MS/MS peaks. False-discovery rate was calculated using Percolator (*42*) and was set to 1% at the peptide level for the whole study. The resulting files were further processed using myProMS (*43*) v.3.9.3 (https://github.com/bioinfo-pf-curie/myproms). Proteins were considered track candidates only if they are (i) never found in PBS samples and (ii) present in both track replicate with at least three peptides in each analysis. The mass spectrometry proteomics data have been deposited to the ProteomeXchange Consortium via the PRIDE [Perez-Riverol *et al.* (*44*)] partner repository with the dataset identifier PXD034091.

### Super-resolution microscopy 3D STORM

For 3D STORM microscopy, immunolabeled cells were prepared on a 1.5 H coverslip using freshly prepared STORM buffer (Smart Kit, Abbelight, Cachan) and sealed with a silicon gasket. The sample was imaged through a 100× 1.49 NA APO TIRF WD 0.13 to 0.20 oil objective lens on a Nikon Ti2 Eclipse (Nikon France SAS, Champigny sur Marne, France) inverted microscope associated to a SAFe 360 detection module (Abbelight, Cachan) combined to two sCMOS Flash 4 v3 cameras (Hamamatsu, Japan). Uniform and large field of view TIRF excitation is provided through an ASTER module (*45*), which included 405 nm (LBX-405-50-CSB-PPA, Oxxius 50 mW) and 640 nm (ERROL laser 500 mW) lasers and a quad band dichroic/emission filter (Semrock 405/488/532/640 refs. FF01-446/510/581/703-25 and Di03-R405/488/532/635-t1-25x36). Acquisition is driven by Neo software (Abbeligth, Cachan) and typically between 20,000 and 40,000 images (20 ms acquisition time) were acquired to reconstruct the final 3D super-resolved images. Two 3D imaging modalities were used. For single protein observation, SAFe detection module was configured for DONALD microscopy (*46*), i.e., the fluorescence image is divided into two imaging paths on the two cameras by a 50-50% beamsplitter, and intrinsic supercritical angle fluorescence (SAF) is used to extract absolute axial position of single-molecule events. For simultaneous dual protein observations labeled with CF647 and CF680, the SAFe module was configured for spectral demixing (*47*), i.e., the fluorescence image is divided into two complementary spectral images by a dichroic with a cutoff wavelength at 700 nm (Chroma T700lpxr-3), and axial information is provided by two astigmatic lens in each imaging path (*48*). 3D STORM analysis including drift correction, axial information calculation (SAF or astigmatism), ratiometric measurements for spectral demixing of CF647 and CF680, and filtering by cluster analysis were performed with Neo analysis software (Abbelight Cachan). Chimerax was repurposed for 3D surface rendering of 3Ddata in movie S3 (*49*).

### Atomic force microscopy

Spectroscopy force experiments were performed in water with an Icon AFM coupled to a Nanoscope V controller from Bruker. The probe was an OTR8 (Bruker) with a spring constant of 0.61 N⋅m. The system was calibrated according to the manufacturer’s procedure: spring constant, deflection sensitivity, and tip size. The approach/retract curves were acquired with a maximum load of 80 N and at a frequency of 1 Hz. The approach/retract curves are processed using software Analysis Nanoscope (Vers. 2.0 Bruker). The moduli represent the average of four values calculated with the Sneddon model on retract curves.

### Statistical analyses

Graphics were prepared with the open-source software Instant Clue (*50*) and Prism V8.0 and statistical analyses were performed using Prism V8.0 or Instant Clue software. Box-plot graphs are built as follows: The line within the box corresponds to the mean value of at least three independent experiments (*n* indicated in each figure legend), boxes indicate interquartile ranges (Q1 to Q3), whiskers extend to min and max values, and, eventually, outliers are represented as points outside the box-whiskers. Empty circles on top of the graphs represent the mean of each individual experiment unless otherwise stated.

## Supplementary Materials

### This PDF file includes:

Figs. S1 to S7 Legends for tables S1 and S2 Legends for movies S1 to S5 References
