# Supplementary material for: Fibroblasts generate topographical cues that steer cancer cell migration
Source: Sci Adv. 2023 Aug 16;9(33):eade2120. doi: 10.1126/sciadv.ade2120 (PMC10431708; doi:10.1126/sciadv.ade2120)
Supplement: Supplementary file 1 — Figs. S1 to S7 Legends for tables S1 and S2 Legends for movies S1 to S5 References [file sciadv.ade2120_sm.pdf]

Science Advances
AAAS

Supplementary Materials for

Fibroblasts generate topographical cues that steer cancer cell migration

Francesco Baschieri *et al.*

Corresponding author: Francesco Baschieri, francesco.baschieri@inserm.fr;
Guillaume Montagnac, guillaume.montagnac@gustaveroussy.fr

Sci. Adv. **9**, eade2120 (2023)
DOI: 10.1126/sciadv.ade2120

The PDF file includes:

Figs. S1 to S7
Legends for tables S1 and S2
Legends for movies S1 to S5
References

Other Supplementary Material for this manuscript includes the following:

Tables S1 and S2
Movies S1 to S5

Supplementary Figure Legends

Supplementary Figure 1. Characterization of CAF-tracks. A, CAAX-mOrange-expressing CAFs were allowed to deposit tracks on glass and tracks were imaged by spinning disk microscopy upon CAFs disconnecting from the tracks. Scale bar: 10 µm. **B**, CAFs migrating on glass were incubated with Calcein AM and imaged by confocal microscopy (left panel) and Interference Reflection Microscopy (right panel). Scale bar: 10 µm. **C**, Representative images of integrin β5-GFP staining in mouse osteoblasts (top left panel) or of αvβ5 integrin staining in the other indicated cell lines. Scale bars: 20 µm. **D**, **E**, CAFs migrating on glass were fixed and stained for β5-integrin (blue) and phalloidin (D, red) or tubulin (E, red). Scale bars: 10 µm. **F**, Super resolution microscopy analyses of β5-integrin-BFP tracks. Scale bars, x-y plane: 10 µm, x-z plane: 100 nm. A color-coded scale indicates the distance from the glass in nm. **G**, Average track tubules diameter in x-y and x-z planes as measured by super resolution microscopy as in B. Data are expressed as mean diameter ± SD.

Supplementary Figure 2. Characterization of tracks integrins content. A, Representative immunofluorescence image of β1-integrin-depleted CAF marked with Alexa-488-labelled Wheat Germ agglutinin. Scale bar: 20 µm. **B**, Western blot analysis of β1-integrin expression in CAFs treated with the indicated siRNAs. Tubulin was used as a loading control. Molecular weights are indicated. **C**, Representative immunofluorescence image of β1-integrin-knockout osteoblasts marked with Alexa-488-labelled Wheat Germ agglutinin. Scale bar: 20 µm. **D**, Western-blot analysis of β1-integrin expression in wild type (WT) or integrin $\beta1^{-/-}$ mouse osteoblasts. Tubulin was used as a loading control. Molecular weights are indicated. **E**, CAFs were plated on Collagen I coated glass and allowed to migrate for 48h before to be fixed and stained for β1 and β5-integrin.

Scale bar: 30 µm. **F**, Representative immunofluorescence images of CAFs treated with the indicated siRNAs and stained for αvβ5 integrin. Scale bar: 30 µm. **G**, Western-blot analysis of β5-integrin expression in CAFs treated with the indicated siRNAs as described in the Materials and Methods. Tubulin was used as a loading control. Molecular weights are indicated. **H**, CAFs treated with the indicated siRNAs were imaged every 20 min for 12 h in the presence or not of 10 µM blebbistatin. Cells were manually tracked and velocity was obtained using the Chemotaxis tool plugin in FIJI software. 459, 676, 188, and 615 cells were tracked from three independent experiments in the control, Integrin β5 siRNA1, Integrin β5 siRNA2, and blebbistatin treated, respectively. Data are expressed as mean velocity ± SD (Kruskal-Wallis test). **I**, CAFs depleted for integrin β5 were transfected with a plasmid encoding for siRNA-resistant integrin β5-GFP and allowed to migrate for 24 h prior to be fixed and stained for integrin β5. Scale bar: 30 µm. The star marks a cell that is not transfected by β5-GFP. **J**, Western blot analysis of YAP expression in CAFs treated with the indicated siRNAs. Tubulin was used as a loading control. Molecular weights are indicated.

Supplementary Figure 3. Analyses of focal adhesions interactions with tracks. A, MDA-MB-231 cells were allowed to spread on CAF-tracks for 35 min before to be fixed and stained for αvβ5-integrin and Talin1. Scale bar: 10 µm. **B**, Quantification of Talin1-associated fluorescence in track areas versus other areas of the plasma membrane in cells as in A. Quantifications were performed with aligned Talin1 and αvβ5-integrin signals (on tracks) as well as upon shifting the αvβ5-integrin signal by 10 pixels (10 pixels shift). 47 cells from 3 independent experiments were analyzed. A value of 1 (blue line) means no enrichment on tracks. Results are represented as mean ratio of track-associated versus non-track-associated Talin1 signal ± SD (Student's T-test). **C**, Western

blot analysis of Talin1 expression in MDA-MB-231 treated with the indicated siRNAs. Tubulin was used as a loading control. Molecular weights are indicated. Of note: according to the technical datasheet, the Talin1 antibody used detects unspecific bands which do not disappear upon Talin1 depletion. **D**, MDA-MB-231 cells transfected with the indicated siRNA were fixed and stained for Talin1. Scale bars: 20 µm. **E**, MDA-MB-231 treated with the indicated siRNAs were allowed to spread for 35 min before to be fixed and stained with phalloidin. Cells on tracks and cells outside tracks were manually segmented and circularity was calculated in FIJI. Results are represented as mean ± SD (Kruskal-Wallis test). **F**, Representative image of MDA-MB-231 transfected with Talin1 siRNA1 and adhering along a track (left) or in regions devoid of tracks (right). Scale bar: 10 µm. **G**, MDA-MB-231 were allowed to spread for 35 min before to be fixed and stained for P-MLC and phalloidin. Cells adhering on tracks and outside tracks were manually segmented and the mean intensity of P-MLC was calculated. Results are expressed as means from 3 independent experiments (no significant difference observed with Student's t-test). **H**, Representative image of MDA-MB-231 cells as in G. The star marks a cell that has adhered on tracks, while the triangle points to a cell adhering outside tracks. Scale bar: 10 µm.

Supplementary Figure 4. Analyses of CCSs interactions with tracks. A, HCT116 cells were allowed to spread on CAF-tracks expressing CAAX-GFP to visualize tracks (blue) for 1 h before to be fixed and stained for and α-adaptin (red). Scale bar: 5 µm. **B**, Quantification of α-adaptin-associated fluorescence in track areas versus other areas of the plasma membrane in cells as in A. Quantifications were performed with aligned α-adaptin and β5-integrin signals (on tracks) as well as upon shifting the β5-integrin signal by 10 pixels. 47 HCT116 cells from 3 independent experiments were analyzed. A value of 1 (blue line) means no enrichment on tracks. Results are represented as mean ratio of track-associated versus non-track-associated α-adaptin signal ± SD

(Student's T-test). **C**, MDA-MB-231 cells genome-edited to express µ2-adaptin-mCherry were allowed to adhere on tracks produced by mouse osteoblasts stably expressing β5-integrin-BFP for 30 minutes. Scale bar: 10 µm. **D**, Quantification of µ2-adaptin-mCherry associated fluorescence in track areas versus other areas of the plasma membrane in cells as in C. Quantifications were performed with aligned µ2-adaptin and β5-integrin signals (on tracks) as well as upon shifting the β5-integrin signal by 10 pixels (10 pixels shift). 29 cells from 3 independent experiments were analyzed. A value of 1 (blue line) means no enrichment on tracks. Results are represented as mean ratio of track-associated versus non-track-associated α-adaptin signal ± SD (Student's T-test). **E**, **F**, **G**, MDA-MB-231 genome-edited to express µ2-adaptin-mCherry were allowed to spread on β5-integrin-BFP osteoblast tracks and imaged every 5 sec for 5 min. A representative kymograph (E), lifetime (F) and nucleation rates (G) of CCSs on tracks vs outside tracks are shown. For lifetime and nucleation, 17 cells from three independent experiments were analyzed. Results are expressed as mean ± SD (Student's T-test). **H**, **I**, Western blot analysis of µ2-adaptin (H) or Clathrin Heavy Chain (I) expression in MDA-MB-231 treated with the indicated siRNAs. Tubulin was used as a loading control. Molecular weights are indicated. **J**, The number of MDA-MB-231 cells adhering in CAF-tracks areas versus in osteoblast-tracks areas of the substrates was measured. Results are expressed as the mean ratio ± SD of cell density on CAF-tracks versus in osteoblast-tracks from three independent experiments (Student's T-test). A value of 1 (blue line) means no enrichment on tracks.

Supplementary Figure 5. Analyses of mechanism of CCSs recruitment on tracks. A, MDA-MB-231 cells genome-edited to express µ2-adaptin-mCherry (red) were allowed to spread on β5-integrin-BFP CAF-tracks (blue) in the presence or not of Cytochalasin D, as indicated, and imaged

every 5 min for 3 hours by spinning disk microscopy. A still frame acquired after 30 min of spreading is shown. Scale bars: 10 µm. **B**, Kymographs corresponding to boxed areas in A. **C**, MDA-MB-231 treated or not with Cytochalasin D were allowed to spread on β5-integrin-BFP tracks and imaged every 10 min for 3 hours. Data represent the evolution over time of the mean β5-integrin-BFP fluorescence intensity ± SD from three independent experiments (Unpaired T test with Welch's correction – $p<0.0001$ as compared to Control). **D**, Western-blot analysis of β1- and β5-integrins expression in CAFs cell lysate versus enriched CAF-tracks fraction. Tubulin was used as a loading control. Molecular weights are indicated. **E**, Quantification of µ2-adaptin-mCherry associated fluorescence in track areas versus other areas of the plasma membrane in MDA-MB-231 cells transfected with the indicated siRNAs. At least 15 cells per condition per experiment were analyzed from 3 independent experiments. A value of 1 (blue line) means no enrichment on tracks. Results are expressed as mean ratio of track-associated versus non-track-associated µ2-adaptin signal ± SD. * $p<0.05$, ** $p<0.01$, *** $p=0.001$, **** $p<0.001$ (uncorrected Fisher's LSD test). **F**, Western blot analysis of Thy1 or MFGE8 expression in CAFs treated with the indicated siRNAs. Tubulin was used as a loading control. Molecular weights are indicated. **G**, CAFs were allowed to migrate on glass prior to be fixed and stained for the indicated proteins. Scale bars: 20 µm.

Supplementary Figure 6. Characterization of the receptor for tracks. A, Western-blot analysis of αv- and β3-integrins expression in MDA-MB-231 and HCT116 cells. Tubulin was used as loading control. Molecular weights are indicated. **B**, Western-blot analysis of αv-integrin expression in MDA-MB-231 treated with the indicated siRNAs. Tubulin was used as a loading control. Molecular weights are indicated. **C**, MDA-MB-231 genome-edited to express µ2-adaptin-mCherry (red) and stably expressing β3-integrin-GFP (yellow) were allowed to spread on β5-

integrin-BFP tracks (cyan) in the presence of 10 µM Cytochalasin D and imaged every 5 min for 12 hours. Still picture at 30 min of spreading is shown. Scale bar 10 µm. **D**, Kymograph corresponding to the boxed area in C. **E**, Quantification of µ2-adaptin-mCherry associated fluorescence in track areas versus other areas of the plasma membrane in MDA-MB-231 cells transfected with the indicated siRNAs. 37, 36, and 27 cells respectively for control, Numb, and DAB2 were analysed in 3 independent experiments. A value of 1 (blue line) means no enrichment on tracks. Results are expressed as mean ratio of track-associated versus non-track-associated µ2-adaptin signal ± SD (Kruskal-Wallis test). **F**, Western-blot analysis of Numb and Dab2 expression in MDA-MB-231 treated with the indicated siRNAs. Tubulin was used as a loading control. Molecular weights are indicated.

Supplementary Figure 7. Analyses of rigidity gradient gels and durotaxis capacities. A, Representation of a glass-bottom dish with a rigidity gradient and image of a 0.1-80 kPa rigidity gradient with fluorescent beads embedded in the stiff part of the gel. Scale bar: 200 µm. **B**, Rigidity-gradient gels were prepared as described in the Materials and Methods. Relative measurements of rigidity were performed by Atomic Force Microscopy (AFM) moving orthogonally to the gradient from one extremity of the gel to the other as if following the red dotted line in A. The region that would normally be imaged in cell migration experiments lies in the grey rectangle. Results are expressed as mean ± SD. **C**, MDA-MB-231 cells were allowed to migrate on a collagen-coated rigidity gradient (0.1-80 kPa), previously conditioned or not by osteoblasts, as indicated. MDA-MB-231 cells were imaged every 20 min for 14 hours. Cells were manually tracked and the forward migration index (FMI) was calculated using the Chemotaxis tool plugin in FIJI software. Values close to 0 (blue line) indicate a random migration. Orientation of the gradient is shown. 350 cells and 541 cells were tracked from three independent experiments, for

the non-conditioned and osteoblast-conditioned conditions, respectively. Data are expressed as mean FMI ± SD (Unpaired Student’s T-test).

Supplementary Movies

Movie 1. CAFs deposit tracks as they migrate. CAF stably expressing the plasma membrane marker CAAX-mOrange was allowed to migrate on glass and imaged by spinning disk microscopy every 30 min for 12 hours. Scale bar: 10 µm.

Movie 2. MDA-MB-231 cells migrate along tracks. MDA-MB-231 cell stably expressing Vinculin-GFP and migrating along CAAX-mOrange-marked tracks and imaged by spinning disk microscopy every 5 min for 9 hours. Scale bar: 10 µm.

Movie 3. CCSs of cancer cells wrap around CAF-tracks. 3D rendering of super resolution microscopy analyzes of CCSs of MDA-MB-231 cells (marked with α-adaptin) aligning along and wrapping around a CAF-track (marked with αvβ5 integrin). Scale bar 500 nm.

Movie 4. Actin dynamics is required for track uptake. MDA-MB-231 genome-edited to express µ2-adaptin-mCherry (Red) were allowed to spread on β5-integrin-BFP tracks (Cyan) in the presence or not of 10 µM Cytochalasin D, as indicated. Images were acquired on a spinning disk microscope every 5 min for 2 hours and 45 min. Upper panels show merge images of µ2-adaptin-mCherry and β5-integrin-BFP channels. Lower panels show β5-integrin-BFP channel only. Scale bar: 10 µm.

Movie 5. β3 integrin in MDA-MB-231 cells accumulate along tracks. MDA-MB-231 genome-edited to express µ2-adaptin-mCherry and stably expressing β3-integrin-GFP were allowed to

spread on β5-integrin-BFP tracks and imaged by TIRF microscopy every 5 min for 2 hours and 45 min. Scale bar: 20 μm.

Supplementary tables

Table 1. List of proteins identified in tracks by mass spectrometry. CAFs were allowed to migrate in tissue culture flasks to generate tracks. Cell bodies were then detached by Calyculin A treatment as described in the Materials and Methods. Finally, half of the flasks were incubated with the integrin β5 inhibitor Cilengitide and the other half with DMSO as a control. PBS was used to recover proteins in both cases. Proteins found in eluates from Cilengitide-treated flasks are listed in the columns labeled as “Track”, while the ”PBS” columns contains the proteins found in the control eluate. Two analyses were performed and only the proteins found exclusively in the “Track” fraction in both replicates (labeled in green) were taken into account for further analysis.

Table 2. List of siRNAs. List of siRNAs used in all figures with target gene name, sequence (when available) and reference.

Supp. Figure 1

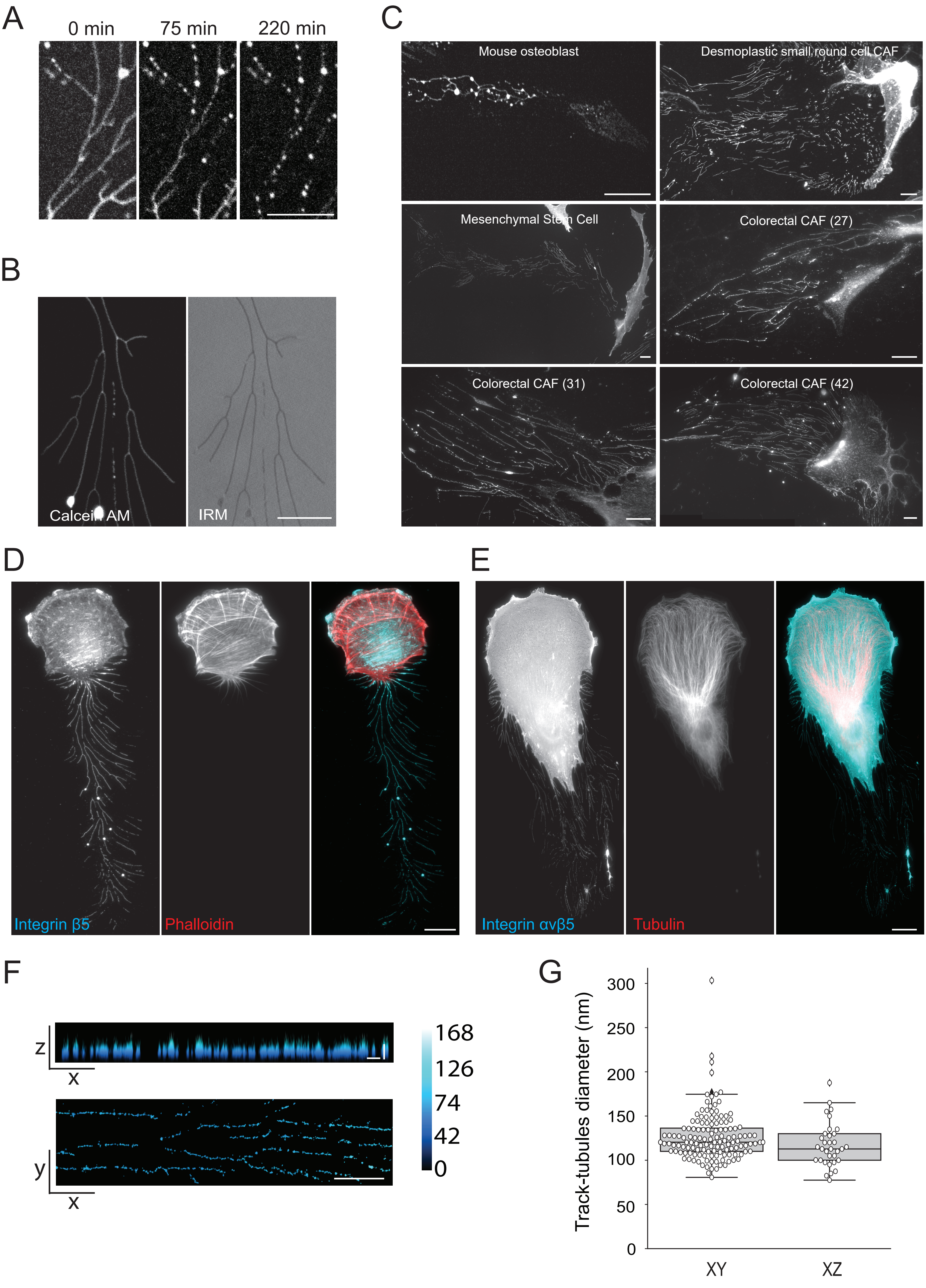

Supp. Figure 2

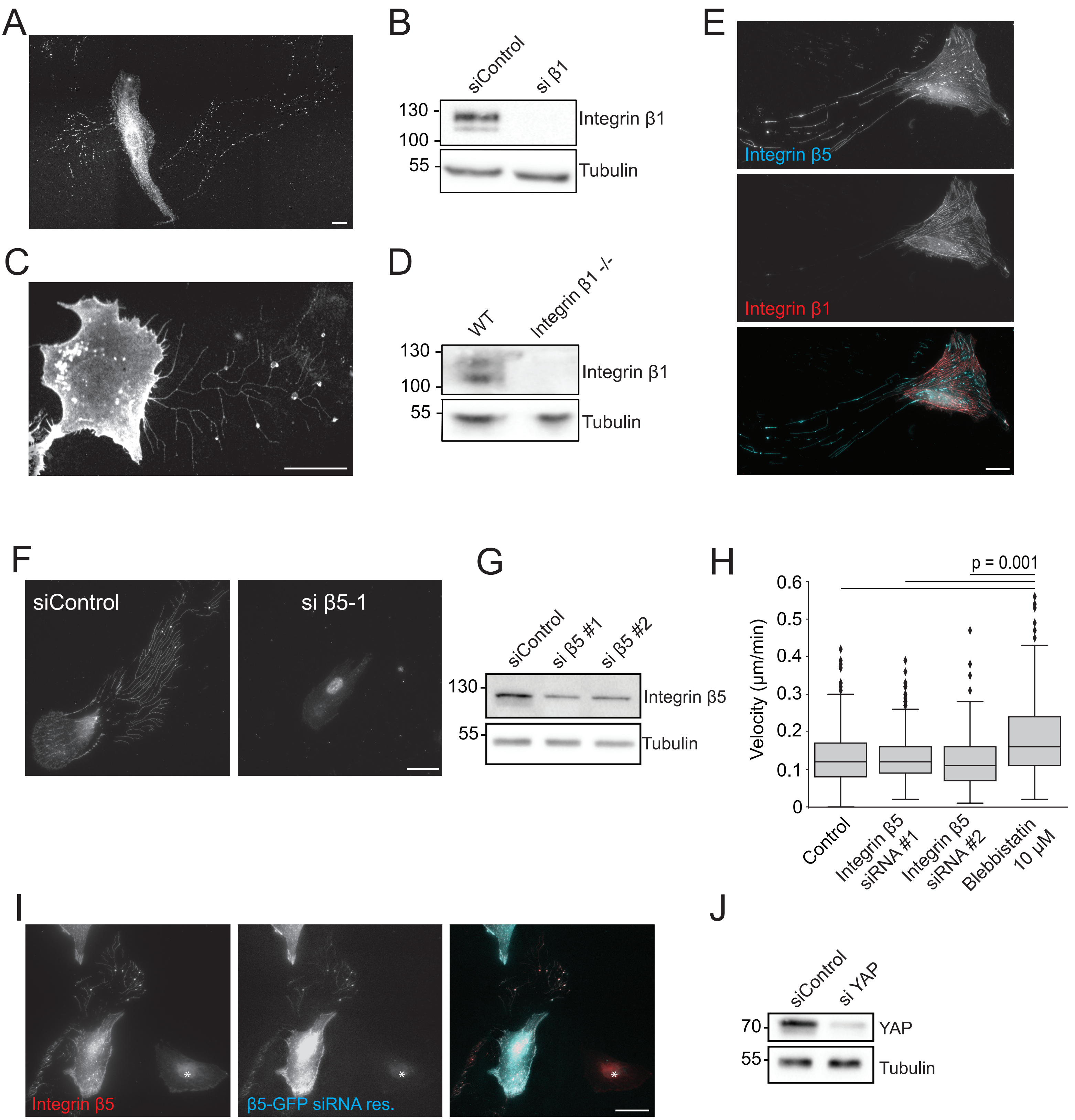

Supp. Figure 3

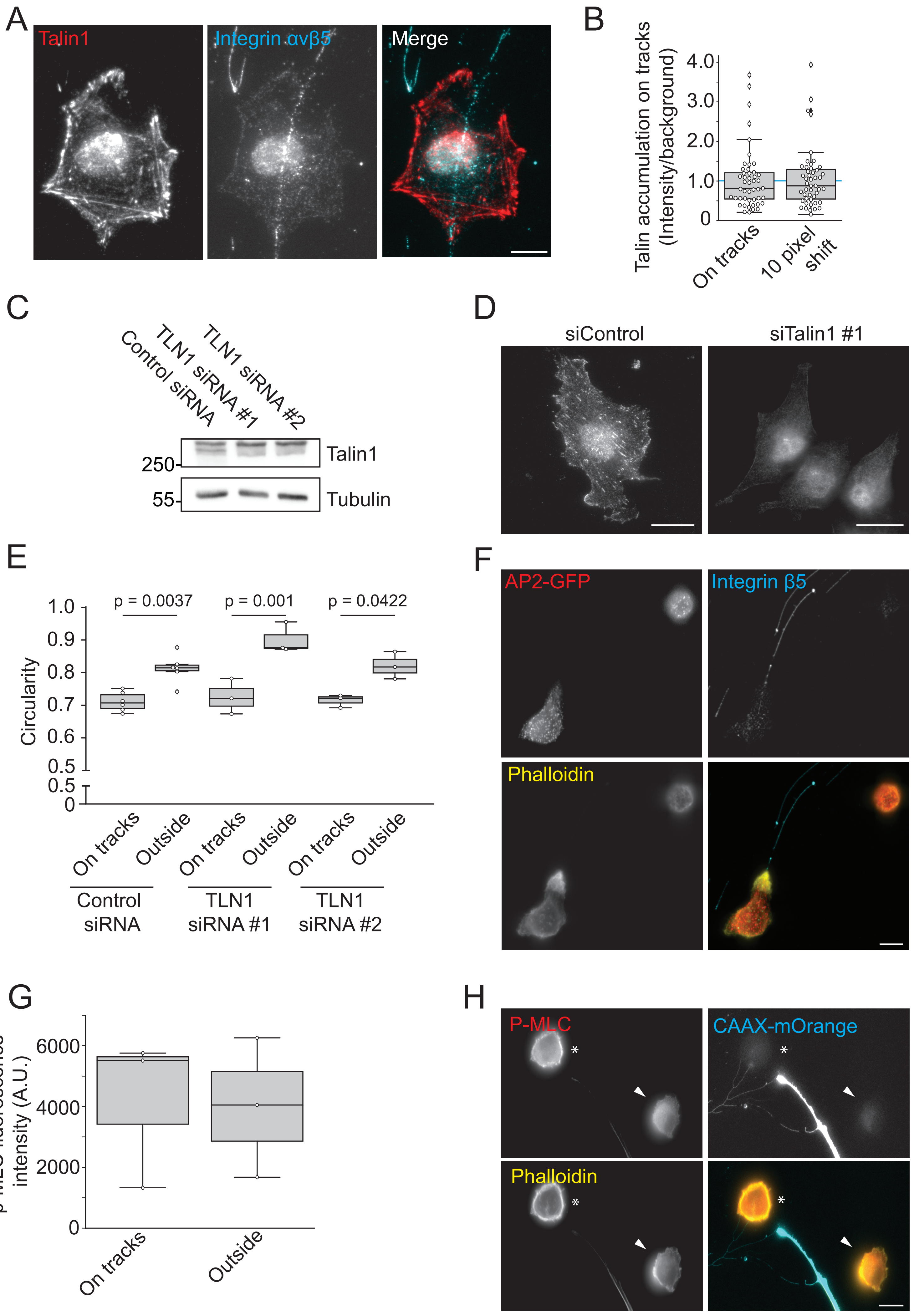

Supp. Figure 4

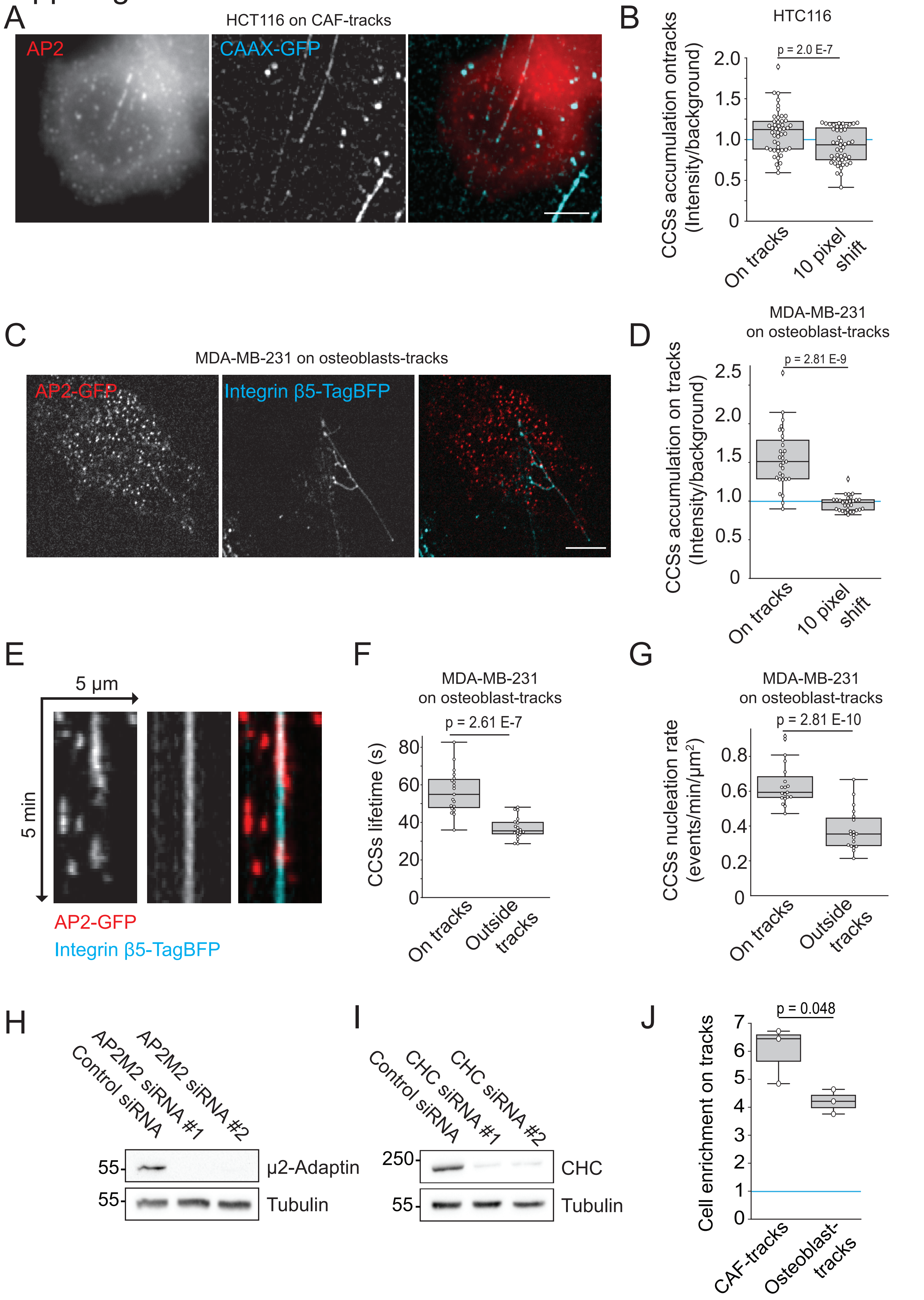

Supp. Figure 5

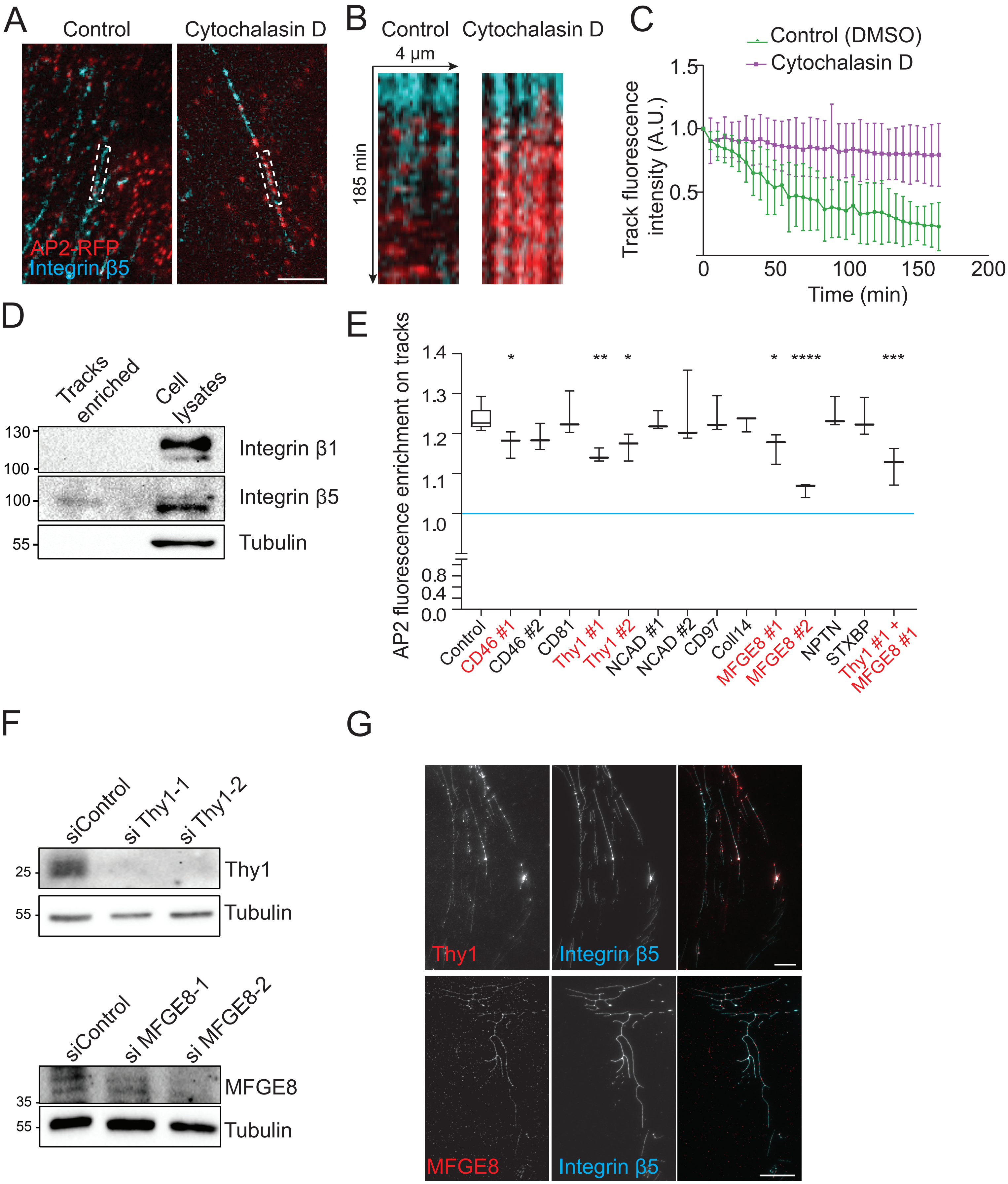

Supp. Figure 6

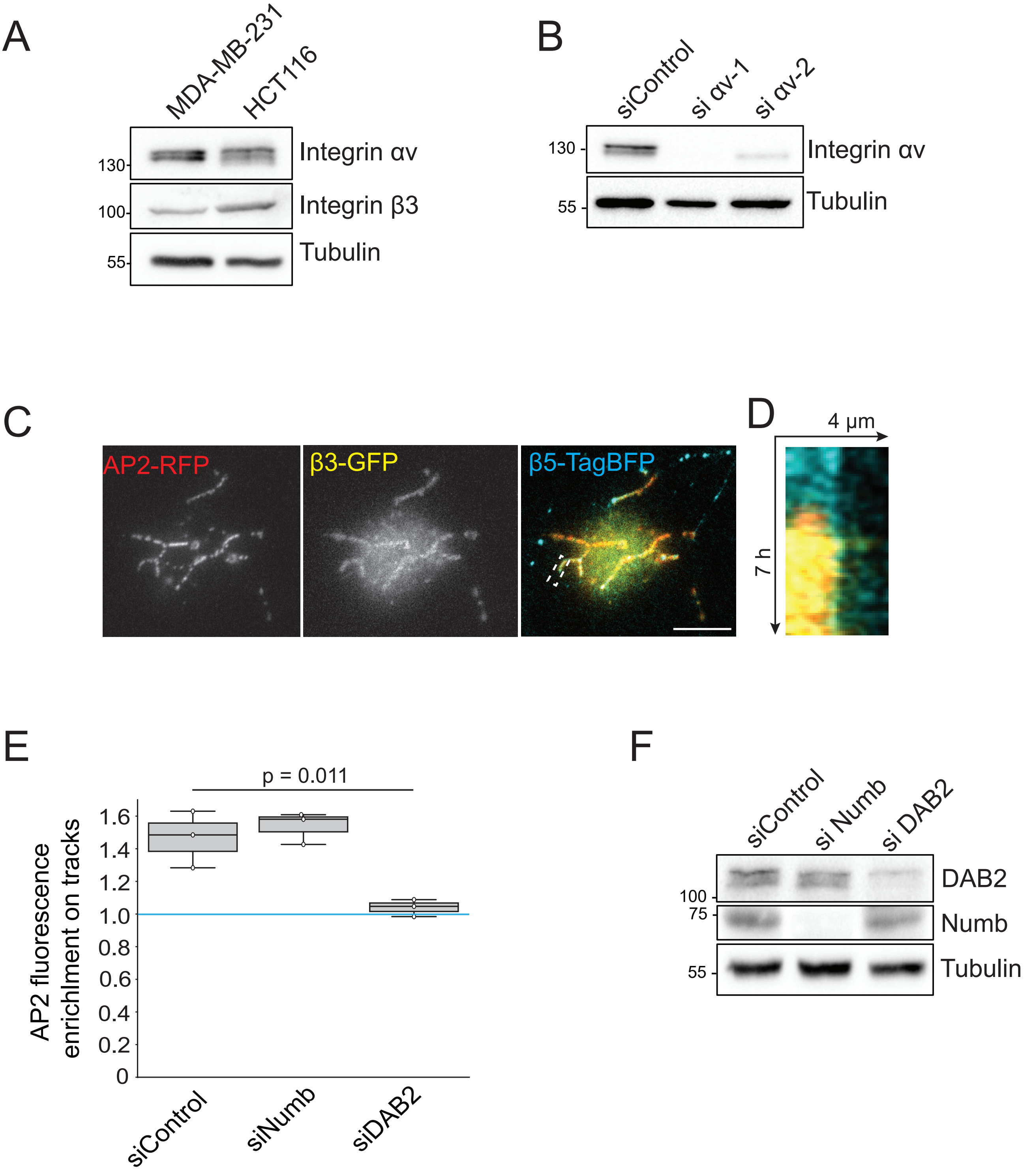

Supp. Figure 7

A

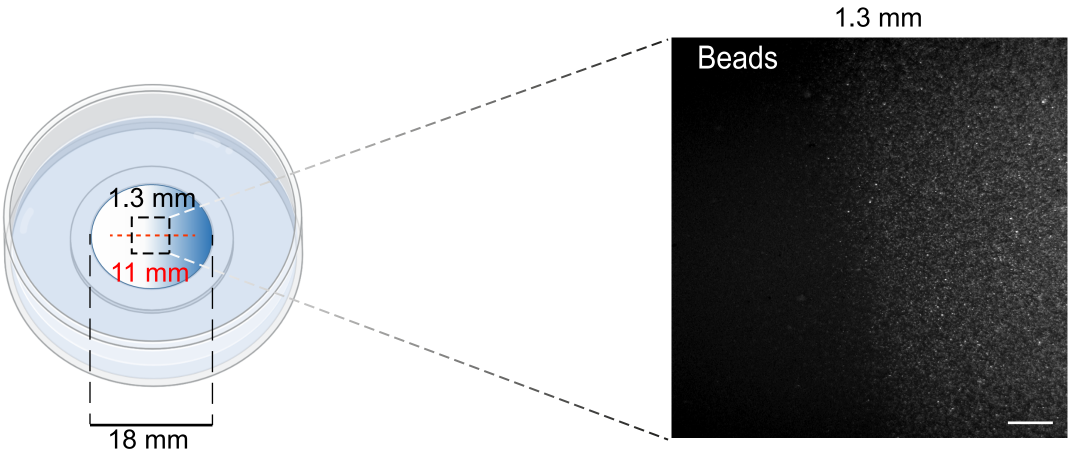

B

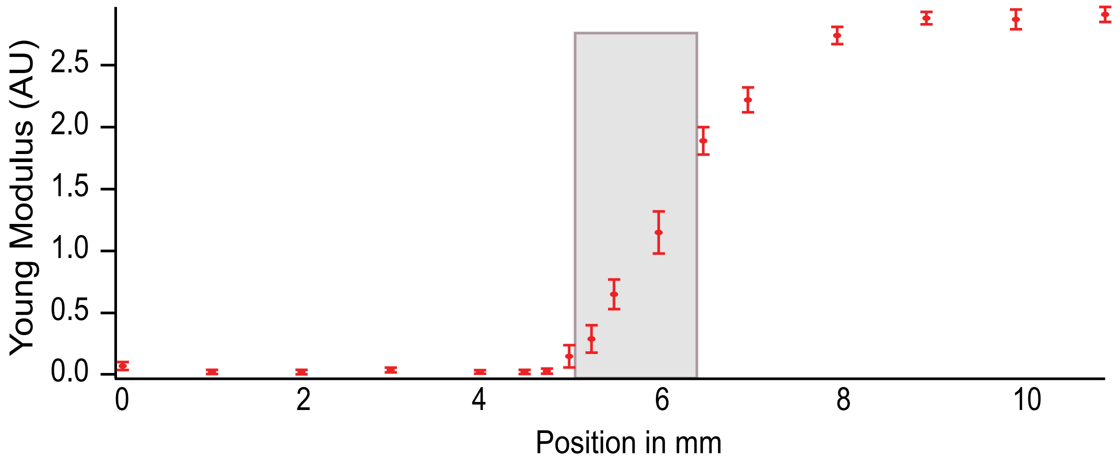

C

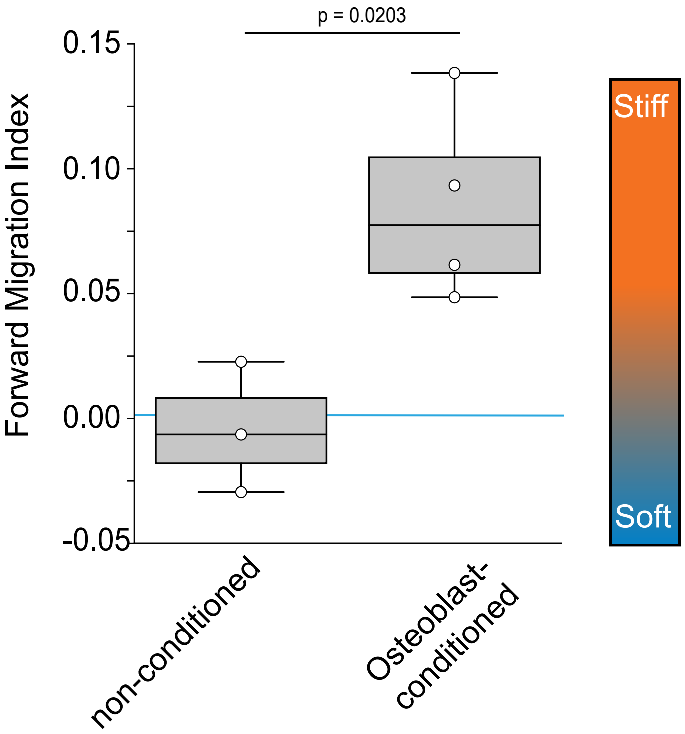

REFERENCES AND NOTES

1. J. Fares, M. Y. Fares, H. H. Khachfe, H. A. Salhab, Y. Fares, Molecular principles of metastasis: A hallmark of cancer revisited. *Signal Trans. Target. Ther.* **5**, 28 (2020).

2. J. Wyckoff, W. Wang, E. Y. Lin, Y. Wang, F. Pixley, E. R. Stanley, T. Graf, J. W. Pollard, J. Segall, J. Condeelis, A paracrine loop between tumor cells and macrophages is required for tumor cell migration in mammary tumors. *Cancer Res.* **64**, 7022–7029 (2004).

3. R. S. Fischer, X. Sun, M. A. Baird, M. J. Hourwitz, B. R. Seo, A. M. Pasapera, S. B. Mehta, W. Losert, C. Fischbach, J. T. Fourkas, C. M. Waterman, Contractility, focal adhesion orientation, and stress fiber orientation drive cancer cell polarity and migration along wavy ECM substrates. *Proc. Natl. Acad. Sci. U.S.A.* **118**, e2021135118 (2021).

4. B. Erdogan, M. Ao, L. M. White, A. L. Means, B. M. Brewer, L. Yang, M. K. Washington, C. Shi, O. E. Franco, A. M. Weaver, S. W. Hayward, D. Li, D. J. Webb, Cancer-associated fibroblasts promote directional cancer cell migration by aligning fibronectin. *J. Cell Biol.* **216**, 3799–3816 (2017).

5. M. J. Oudin, V. M. Weaver, Physical and chemical gradients in the tumor microenvironment regulate tumor cell invasion, migration, and metastasis. *Cold Spring Harb. Symp. Quant. Biol.* **81**, 189–205 (2016).

6. J. Zhang, L. Chen, X. Liu, T. Kammertoens, T. Blankenstein, Z. Qin, Fibroblast-specific protein 1/S100A4-positive cells prevent carcinoma through collagen production and encapsulation of carcinogens. *Cancer Res.* **73**, 2770–2781 (2013).

7. Y. Attieh, A. G. Clark, C. Grass, S. Richon, M. Pocard, P. Mariani, N. Elkhatib, T. Betz, B. Gurchenkov, D. M. Vignjevic, Cancer-associated fibroblasts lead tumor invasion through integrin-β3-dependent fibronectin assembly. *J. Cell Biol.* **216**, 3509–3520 (2017).

8. C. Gaggioli, S. Hooper, C. Hidalgo-Carcedo, R. Grosse, J. F. Marshall, K. Harrington, E. Sahai, Fibroblast-led collective invasion of carcinoma cells with differing roles for RhoGTPases in leading and following cells. *Nat. Cell Biol.* **9**, 1392–1400 (2007).

9. A. Labernadie, T. Kato, A. Brugués, X. Serra-Picamal, S. Derzsi, E. Arwert, A. Weston, V. González-Tarragó, A. Elosegui-Artola, L. Albertazzi, J. Alcaraz, P. Roca-Cusachs, E. Sahai, X. Trepat, A mechanically active heterotypic E-cadherin/N-cadherin adhesion enables fibroblasts to drive cancer cell invasion. *Nat. Cell Biol.* **19**, 224–237 (2017).

10. G. Fuhr, E. Richter, H. Zimmermann, H. Hitzler, H. Niehus, R. Hagedorn, Cell traces—Footprints of individual cells during locomotion and adhesion. *Biol. Chem.* **379**, 1161–1173 (1998).

11. H. Zimmermann, E. Richter, C. Reichle, I. Westphal, P. Geggier, U. Rehn, S. Rogaschewski, W. Bleiss, G. R. Fuhr, Mammalian cell traces—Morphology, molecular composition, artificial guidance and biotechnological relevance as a new type of "bionanotube". *Appl. Phys. A* **73**, 11–26 (2001).

12. L. Ma, Y. Li, J. Peng, D. Wu, X. Zhao, Y. Cui, L. Chen, X. Yan, Y. Du, L. Yu, Discovery of the migrasome, an organelle mediating release of cytoplasmic contents during cell migration. *Cell Res.* **25**, 24–38 (2015).

13. D. Jiang, Z. Jiang, D. Lu, X. Wang, H. Liang, J. Zhang, Y. Meng, Y. Li, D. Wu, Y. Huang, Y. Chen, H. Deng, Q. Wu, J. Xiong, A. Meng, L. Yu, Migrasomes provide regional cues for organ morphogenesis during zebrafish gastrulation. *Nat. Cell Biol.* **21**, 966–977 (2019).

14. Y. Huang, B. Zucker, S. Zhang, S. Elias, Y. Zhu, H. Chen, T. Ding, Y. Li, Y. Sun, J. Lou, M. M. Kozlov, L. Yu, Migrasome formation is mediated by assembly of micron-scale tetraspanin macrodomains. *Nat. Cell Biol.* **21**, 991–1002 (2019).

15. S. Yu, L. Yu, Migrasome biogenesis and functions. *FEBS J.* **289**, 7246–7254 (2022).

16. C. Fan, X. Shi, K. Zhao, L. Wang, K. Shi, Y. J. Liu, H. Li, B. Ji, Y. Jiu, Cell migration orchestrates migrasome formation by shaping retraction fibers. *J. Cell Biol.* **221**, e202109168 (2022).

17. R. Bar-Ziv, E. Moses, Instability and "Pearling" states produced in tubular membranes by competition of curvature and tension. *Phys. Rev. Lett.* **73**, 1392–1395 (1994).

18. H. B. Schiller, M. R. Hermann, J. Polleux, T. Vignaud, S. Zanivan, C. C. Friedel, Z. Sun, A. Raducanu, K. E. Gottschalk, M. Théry, M. Mann, R. Fässler, β1- and αv-class integrins cooperate to regulate

myosin II during rigidity sensing of fibronectin-based microenvironments. *Nat. Cell Biol.* **15**, 625–636 (2013).

19. A. Elosegui-Artola, E. Bazellières, M. D. Allen, I. Andreu, R. Oria, R. Sunyer, J. J. Gomm, J. F. Marshall, J. L. Jones, X. Trepat, P. Roca-Cusachs, Rigidity sensing and adaptation through regulation of integrin types. *Nat. Mater.* **13**, 631–637 (2014).

20. F. Baschieri, S. Dayot, N. Elkhatib, N. Ly, A. Capmany, K. Schauer, T. Betz, D. M. Vignjevic, R. Poincloux, G. Montagnac, Frustrated endocytosis controls contractility-independent mechanotransduction at clathrin-coated structures. *Nat. Commun.* **9**, 3825 (2018).

21. N. Elkhatib, E. Bresteau, F. Baschieri, A. L. Rioja, G. Van Niel, S. Vassilopoulos, G. Montagnac, Tubular clathrin/AP-2 lattices pinch collagen fibers to support 3D cell migration. *Science* **356**, eaal4713 (2017).

22. E. Bresteau, N. Elkhatib, F. Baschieri, K. Bellec, M. Guérin, G. Montagnac, Clathrin-coated structures support 3D directed migration through local force transmission. *Sci. Adv.* **7**, eabf4647 (2021).

23. S. Boulant, C. Kural, J.-C. Zeeh, F. Ubelmann, T. Kirchhausen, Actin dynamics counteract membrane tension during clathrin-mediated endocytosis. *Nat. Cell Biol.* **13**, 1124–1131 (2011).

24. M. Kaksonen, C. P. Toret, D. G. Drubin, Harnessing actin dynamics for clathrin-mediated endocytosis. *Nat. Rev. Mol. Cell Biol.* **7**, 404–414 (2006).

25. D. Perrais, C. J. Merrifield, Dynamics of endocytic vesicle creation. *Dev. Cell* **9**, 581–592 (2005).

26. F. Baschieri, K. Porshneva, G. Montagnac, Frustrated clathrin-mediated endocytosis—Causes and possible functions. *J. Cell Sci.* **133**, jcs240861 (2020).

27. M. Jin, C. Shirazinejad, B. Wang, A. Yan, J. Schöneberg, S. Upadhyayula, K. Xu, D. G. Drubin, Branched actin networks are organized for asymmetric force production during clathrin-mediated endocytosis in mammalian cells. *Nat. Commun.* **13**, 3578 (2022).

28. R. Hanayama, M. Tanaka, K. Miwa, A. Shinohara, A. Iwamatsu, S. Nagata, Identification of a factor

that links apoptotic cells to phagocytes. *Nature* **417**, 182–187 (2002).

29. T. Hermosilla, D. Muñoz, R. Herrera-Molina, A. Valdivia, N. Muñoz, S.-U. Nham, P. Schneider, K. Burridge, A. F. G. Quest, L. Leyton, Direct Thy-1/αVβ3 integrin interaction mediates neuron to astrocyte communication. *Biochim. Biophys. Acta* **1783**, 1111–1120 (2008).

30. L. Leyton, P. Schneider, C. V. Labra, C. Rüegg, C. A. Hetz, A. F. G. Quest, C. Bron, Thy-1 binds to integrin β3 on astrocytes and triggers formation of focal contact sites. *Curr. Biol.* **11**, 1028–1038 (2001).

31. C.-h Yu, N. B. M. Rafiq, F. Cao, Y. Zhou, A. Krishnasamy, K. H. Biswas, A. Ravasio, Z. Chen, Y.-H. Wang, K. Kawauchi, G. E. Jones, M. P. Sheetz, Integrin-beta3 clusters recruit clathrin-mediated endocytic machinery in the absence of traction force. *Nat. Commun.* **6**, 8672 (2015).

32. C. M. Lo, H. B. Wang, M. Dembo, Y. L. Wang, Cell movement is guided by the rigidity of the substrate. *Biophys. J.* **79**, 144–152 (2000).

33. S. V. Plotnikov, A. M. Pasapera, B. Sabass, C. M. Waterman, Force fluctuations within focal adhesions mediate ECM-rigidity sensing to guide directed cell migration. *Cell* **151**, 1513–1527 (2012).

34. B. J. DuChez, A. D. Doyle, E. K. Dimitriadis, K. M. Yamada, Durotaxis by human cancer cells. *Biophys. J.* **116**, 670–683 (2019).

35. J. G. Lock, F. Baschieri, M. C. Jones, J. D. Humphries, G. Montagnac, S. Strömblad, M. J. Humphries, Clathrin-containing adhesion complexes. *J. Cell Biol.* **218**, 2086–2095 (2019).

36. J. G. Lock, M. C. Jones, J. A. Askari, X. Gong, A. Oddone, H. Olofsson, S. Göransson, M. Lakadamyali, M. J. Humphries, S. Strömblad, Reticular adhesions are a distinct class of cell-matrix adhesions that mediate attachment during mitosis. *Nat. Cell Biol.* **20**, 1290–1302 (2018).

37. B. H. Sung, A. von Lersner, J. Guerrero, E. S. Krystofiak, D. Inman, R. Pelletier, A. Zijlstra, S. M. Ponik, A. M. Weaver, A live cell reporter of exosome secretion and uptake reveals pathfinding behavior of migrating cells. *Nat. Commun.* **11**, 2092 (2020).

38. J. d'Alessandro, A. Barbier--Chebbah, V. Cellerin, O. Benichou, R. M. Mège, R. Voituriez, B. Ladoux,

Cell migration guided by long-lived spatial memory. *Nat. Commun.* **12**, 4118 (2021).

39. C. Huet-Calderwood, F. Rivera-Molina, D. V. Iwamoto, E. B. Kromann, D. Toomre, D. A. Calderwood, Novel ecto-tagged integrins reveal their trafficking in live cells. *Nat. Commun.* **8**, 570 (2017).

40. T. Betz, D. Koch, Y. B. Lu, K. Franze, J. A. Käs, Growth cones as soft and weak force generators. *Proc. Natl. Acad. Sci. U.S.A.* **108**, 13420–13425 (2011).

41. A. M. Cozzolino, V. Noce, C. Battistelli, A. Marchetti, G. Grassi, C. Cicchini, M. Tripodi, L. Amicone, Modulating the substrate stiffness to manipulate differentiation of resident liver stem cells and to improve the differentiation state of hepatocytes. *Stem Cells Int.* **2016**, 5481493 (2016).

42. M. The, M. J. MacCoss, W. S. Noble, L. Käll, Fast and accurate protein false discovery rates on large-scale proteomics data sets with Percolator 3.0. *J. Am. Soc. Mass Spectrom.* **27**, 1719–1727 (2016).

43. P. Poullet, S. Carpentier, E. Barillot, myProMS, a web server for management and validation of mass spectrometry-based proteomic data. *Proteomics* **7**, 2553–2556 (2007).

44. Y. Perez-Riverol, A. Csordas, J. Bai, M. Bernal-Llinares, S. Hewapathirana, D. J. Kundu, A. Inuganti, J. Griss, G. Mayer, M. Eisenacher, E. P érez, J. Uszkoreit, J. Pfeuffer, T. Sachsenberg, S. Yilmaz, S. Tiwary, J. Cox, E. Audain, M. Walzer, A. F. Jarnuczak, T. Ternent, A. Brazma, J. A. Vizcaíno, The PRIDE database and related tools and resources in 2019: Improving support for quantification data. *Nucleic Acids Res.* **47**, D442–D450 (2019).

45. A. Mau, K. Friedl, C. Leterrier, N. Bourg, S. Lévêque-Fort, Fast widefield scan provides tunable and uniform illumination optimizing super-resolution microscopy on large fields. *Nat. Commun.* **12**, 3077 (2021).

46. N. Bourg, C. Mayet, G. Dupuis, T. Barroca, P. Bon, S. Lécart, E. Fort, S. Lévêque-Fort, Direct optical nanoscopy with axially localized detection. *Nat. Photonics* **9**, 587–593 (2015).

47. A. Lampe, V. Haucke, S. J. Sigrist, M. Heilemann, J. Schmoranzer, Multi-colour direct STORM with red emitting carbocyanines. *Biol. Cell* **104**, 229–237 (2012).

48. C. Cabriel, N. Bourg, P. Jouchet, G. Dupuis, C. Leterrier, A. Baron, M.-A. Badet-Denisot, B. Vauzeilles, E. Fort, S. Lévêque-Fort, Combining 3D single molecule localization strategies for reproducible bioimaging. *Nat. Commun.* **10**, 1980 (2019).

49. E. F. Pettersen, T. D. Goddard, C. C. Huang, E. C. Meng, G. S. Couch, T. I. Croll, J. H. Morris, T. E. Ferrin, UCSF ChimeraX: Structure visualization for researchers, educators, and developers. *Protein Sci.* **30**, 70–82 (2021).

50. H. Nolte, T. D. MacVicar, F. Tellkamp, M. Krüger, Instant clue: A software suite for interactive data visualization and analysis. *Sci. Rep.* **8**, 12648 (2018).

51. E. Tejera, V. Rocha-Perugini, S. López-Martín, D. Pérez-Hernández, A. I. Bachir, A. R. Horwitz, J. Vázquez, F. Sánchez-Madrid, M. Yáñez-Mo, CD81 regulates cell migration through its association with Rac GTPase. *Mol. Biol. Cell* **24**, 261–273 (2013).

52. M. S. Rezcallah, K. Hodges, D. B. Gill, J. P. Atkinson, B. Wang, P. P. Cleary, Engagement of CD46 and α5β1 integrin by group A streptococci is required for efficient invasion of epithelial cells. *Cell. Microbiol.* **7**, 645–653 (2005).

53. S. Zhang, C. Shan, W. Cui, X. You, Y. Du, G. Kong, F. Gao, L. Ye, X. Zhang, Hepatitis B virus X protein protects hepatoma and hepatic cells from complement-dependent cytotoxicity by up-regulation of CD46. *FEBS Lett.* **587**, 645–651 (2013).

54. Q. Li, A. R. Wilkie, M. Weller, X. Liu, J. I. Cohen, THY-1 cell surface antigen (CD90) has an important role in the initial stage of human cytomegalovirus infection. *PLOS Pathog.* **11**, e1004999 (2015).

55. A. Labernadie, T. Kato, A. Brugués, X. Serra-Picamal, S. Derzsi, E. Arwert, A. Weston, V. González-Tarragó, A. Elosegui-Artola, L. Albertazzi, J. Alcaraz, P. Roca-Cusachs, E. Sahai, X. Trepat, A mechanically active heterotypic E-cadherin/N-cadherin adhesion enables fibroblasts to drive cancer cell invasion. *Nat. Cell Biol.* **19**, 224–237 (2017).

56. B. Wang, Z. Ge, Y. Wu, Y. Zha, X. Zhang, Y. Yan, Y. Xie, MFGE8 is down-regulated in cardiac fibrosis and attenuates endothelial-mesenchymal transition through Smad2/3-Snail signalling pathway.

J. Cell. Mol. Med. **24**, 12799–12812 (2020).
